# MOV10 inhibits SADS-CoV replication by enhancing TRIM24-mediated K63-linked TRAF3 ubiquitination, and this inhibition is antagonized by viral N protein

**DOI:** 10.1128/jvi.01215-26

**Published:** 2026-09-29

**Authors:** Miaomiao Zeng, Dakai Liu, Jiyu Zhang, Liaoyuan Zhang, Hongyan Shi, Xin Zhang, Jianfei Chen, Xiuwen Li, Jialin Zhang, Tingshuai Feng, Xinwei Sun, Junyi Su, Zhaoyang Ji, Li Feng, Da Shi

**Affiliations:** 1 State Key Laboratory of Harbin Veterinary Research Institute, Chinese Academy of Agricultural Sciences, Harbin, China; Loyola University Chicago - Health Sciences Campus, Maywood, Illinois, USA

**Keywords:** SADS-CoV, MOV10, TRAF3, ubiquitination, TRIM24, N protein

## Abstract

The global outbreak of SARS-CoV-2 has resulted in a renewed focus on coronaviruses with the potential for cross-species transmission and mutation risks. In particular, swine acute diarrhea syndrome coronavirus (SADS-CoV), potentially originating from the intermediate horseshoe bat (*Rhinolophus affinis*), is a novel coronavirus that causes acute diarrhea, vomiting, and high mortality in suckling piglets. Innate immunity plays a crucial role in defending the host against invading pathogens. Previous studies have identified several host factors that inhibit SADS-CoV replication through innate immunity; however, research on SADS-CoV remains insufficient compared to that on other coronaviruses. This study demonstrates that Moloney leukemia virus 10 protein (MOV10) exerts anti-SADS-CoV activity via its N-terminal domain in a helicase activity-independent manner. Mechanistic analysis reveals that MOV10 induces translocation of tripartite motif-containing 24 (TRIM24) from the nucleus to the cytoplasm. The cytoplasmically localized TRIM24 binds to MOV10 and TNF receptor-associated factor 3 (TRAF3) to form a ternary complex. Functional studies demonstrate that MOV10 strengthens the interaction between TRIM24 and TRAF3 in a dose-dependent manner and facilitates TRIM24-mediated K63-linked ubiquitination of TRAF3, thereby activating interferon immune responses. Furthermore, we identify the strategies by which SADS-CoV evades innate immunity. The viral N protein inhibits the antiviral effect of MOV10 by disrupting the interaction between MOV10 and TRAF3 and by promoting K48-linked polyubiquitination of TRAF3, leading to TRAF3 degradation via the ubiquitin-proteasome pathway. Collectively, our findings reveal the role of MOV10 in antiviral immunity and how SADS-CoV evades host immune defense.

**IMPORTANCE:**

Moloney leukemia virus 10 protein (MOV10), a DExD-box RNA helicase, is a known component of cytoplasmic RNA processing centers known as P bodies. As an interferon-stimulated gene (ISG), MOV10 exhibits broad-spectrum antiviral activity against multiple viruses, including SARS-CoV-2, HIV, HSV-1, IAV, and PRRSV. Here, we identify a novel helicase-independent antiviral mechanism of MOV10 against SADS-CoV. In this study, we show that MOV10 bridges TRIM24 and TRAF3 and facilitates K63-linked ubiquitination of TRAF3 independent of its helicase activity. This process strongly amplifies downstream interferon antiviral responses and suppresses viral replication. Furthermore, the SADS-CoV N protein efficiently antagonizes MOV10-mediated immune defense by disrupting the MOV10-TRAF3 interaction and inducing K48-linked ubiquitination and proteasomal degradation of TRAF3. Collectively, this study establishes a previously uncharacterized MOV10-TRIM24-TRAF3 antiviral regulatory axis, which offers new mechanistic insights into how coronaviruses evade host immune defenses.

## INTRODUCTION

Circulating coronaviruses in wildlife populations pose a substantial public health threat due to their zoonotic potential. Based on genetic and phenotypic characteristics, coronaviruses are classified into four distinct genera: *Alphacoronavirus*, *Betacoronavirus*, *Gammacoronavirus*, and *Deltacoronavirus*. In general, the former two infect only mammals, while the latter two mostly infect birds, but some of them can also infect mammals (1). Several alphacoronaviruses, such as porcine epidemic diarrhea virus (PEDV), porcine transmissible gastroenteritis virus (TGEV), and swine acute diarrhea syndrome coronavirus (SADS-CoV), cause significant gastroenteritis in neonatal piglets (2).

SADS-CoV is a novel coronavirus first identified in China in 2017 (3). The clinical signs of SADS-CoV infection in piglets include acute diarrhea, vomiting, and a mortality rate approaching 90% (4, 5). Since its initial discovery, SADS-CoV has caused outbreaks in multiple provinces across China (4, 6), resulting in significant economic losses. SADS-CoV has recently been detected in swine populations in Vietnam, indicating that the virus has spread there from China (7). This suggests that the virus has extended geographically, warranting global attention. The nucleocapsid (N) protein has the highest abundance in infected cells and is essential for viral transcription and assembly (8, 9). In addition to viral function, the SADS-CoV N protein is considered an important interferon (IFN) antagonist (10). For example, it performs this role during infection by severe acute respiratory syndrome coronavirus 2 (SARS-CoV-2) (11), severe acute respiratory syndrome coronavirus (SARS-CoV) (12), mouse hepatitis virus (MHV) (13), porcine deltacoronavirus (PDCoV) (14), and PEDV (15). Although SADS-CoV has been shown to inhibit IFN-β production (10, 16), the roles of its proteins in evading antiviral immunity and the underlying molecular mechanisms remain unclear.

Moloney leukemia virus 10 protein (MOV10), a 110-kDa RNA helicase, is a known component of cytoplasmic RNA processing centers called P-bodies (17). Its helicase activity unwinds RNA in an ATP-dependent manner and in a 5′ to 3′ direction (18). Additionally, MOV10 is an IFN-stimulated gene (ISG) (19, 20) that plays a crucial role in the innate immune response. During viral infection, viral RNA is recognized by host RNA sensors, such as retinoic acid inducible gene I (RIG-I), which triggers production of IFN via the MAVS-TRAF3-TBK1-IRF3 signaling pathway (21). The activated IFN response further upregulates MOV10 expression, which endows MOV10 with broad-spectrum antiviral activity against diverse RNA viruses. Although different RNA viruses evolve multiple distinct strategies to antagonize host IFN responses (22), MOV10 is capable of efficiently restricting a wide range of RNA viruses regardless of their immune evasion mechanisms. Ubiquitination, a key post-translational modification, plays a critical role in regulating the host IFN signaling pathway (23). Upon RNA virus infection, the signaling molecules in this pathway, such as RIG-I, mitochondrial antiviral signaling protein (MAVS; also known as IPS-1, VISA, and CARDIF), and TRAF3, undergo different types of ubiquitination by various E3 ubiquitin ligases and thus have different outcomes (24). For example, K48-linked ubiquitination guides these molecules for proteasome degradation (24), whereas K63-linked ubiquitination promotes activation of downstream signaling and enhances transcription of IFN-α/β (25). The balance between these opposing ubiquitination pathways generates a negative feedback loop to restrain IFN-I signaling. Consequently, activation of the IFN-I signaling pathway is finely tuned by dynamic regulation of the ubiquitination status of these signaling molecules by E3 ligases and deubiquitinating enzymes.

In this study, we found that SADS-CoV infection induced expression of MOV10 *in vitro* and *in vivo*. MOV10 inhibited SADS-CoV replication mainly through its N-terminal domain. We also demonstrated that MOV10 enhanced the IFN-β response during SADS-CoV infection, with TRAF3 playing a critical role in the MOV10-regulated innate immune pathway. Additionally, MOV10 promoted K63-linked polyubiquitination of TRAF3 mediated by TRIM24. Furthermore, we investigated how SADS-CoV evaded the host immune system. The antiviral function of MOV10 was counteracted by the viral N protein, which disrupted the interaction of MOV10 with TRAF3, impairing the IFN signaling pathway. The N protein also directly targeted TRAF3 for K48-linked ubiquitination and degradation, inhibiting IFN production during the later stages of SADS-CoV infection. In summary, our findings highlighted the essential antiviral role of MOV10 in SADS-CoV infection and revealed how SADS-CoV manipulated the IFN signaling pathway to evade the innate immune response. These insights provide a deeper understanding of the complex interaction between viral evasion strategies and host immune defenses.

## RESULTS

### Induction of MOV10 expression by SADS-CoV infection *in vitro* and *in vivo*

**Fig 1:**
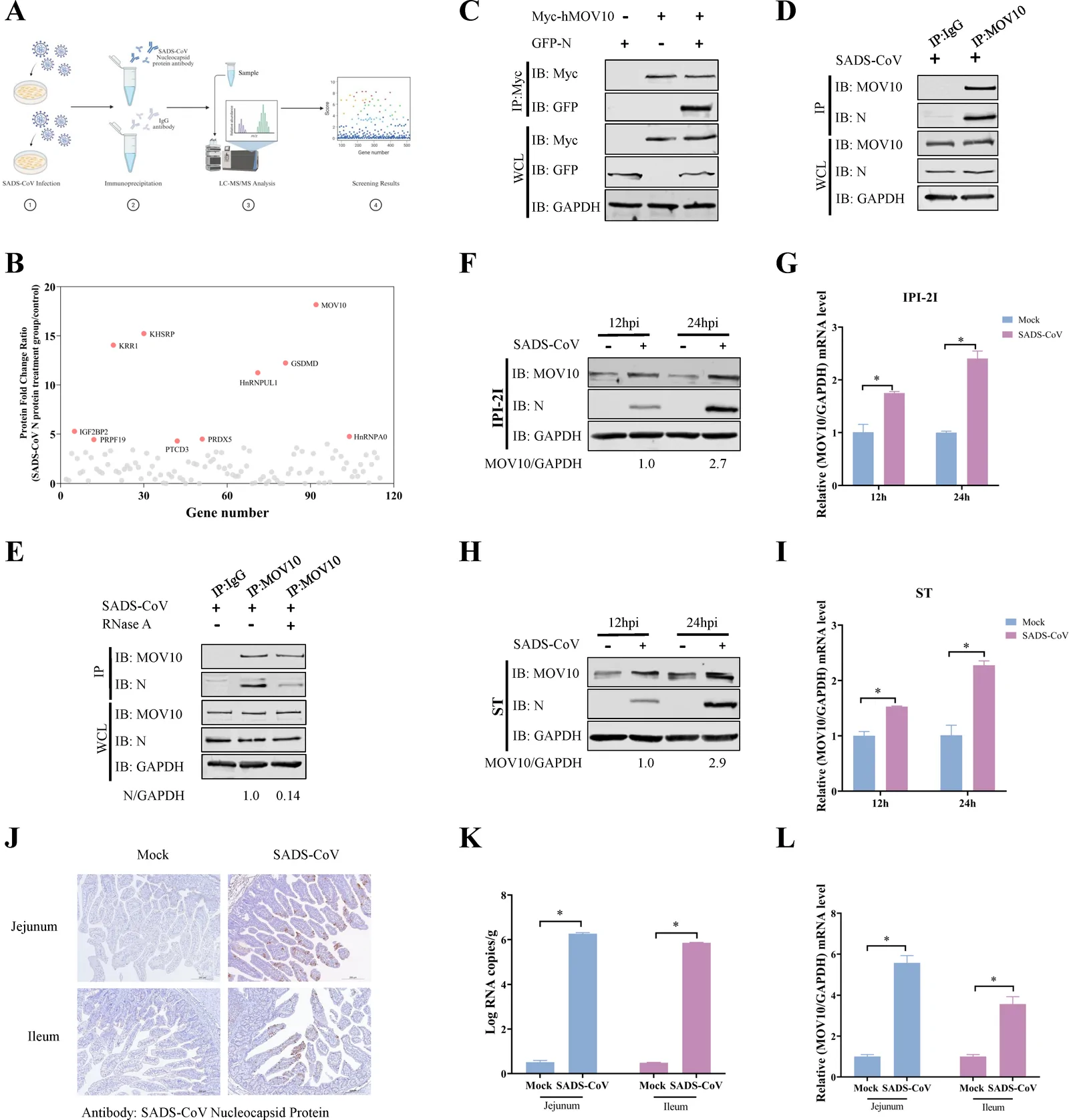
Induction of MOV10 expression by SADS-CoV infection *in vitro* and *in vivo*. (**A**) Flowchart illustrating the identification of host factors associated with SADS-CoV N protein. (**B**) Fold change in protein expression (y axis) and gene number (x axis) of host factors identified in Vero E6 cells infected with SADS-CoV. (**C**) MOV10 interacted with the SADS-CoV N protein. HEK293T cells were transfected or co-transfected with plasmids encoding Myc-hMOV10 and GFP-N. Cell lysates were subjected to immunoprecipitation using Myc Abs. (**D**) Endogenous MOV10 interacted with the SADS-CoV N protein in IPI-2I cells. IPI-2I cells were infected with SADS-CoV (MOI = 0.1) for 24 h. Lysates were subjected to immunoprecipitation using MOV10 Ab, with IgG Ab as the negative control. Immunoprecipitants were analyzed by western blotting using Abs against MOV10, SADS-CoV N, and GAPDH. (**E**) Interaction between SADS-CoV N and MOV10 was likely RNA dependent. IPI-2I cells were infected with SADS-CoV (MOI = 0.1) for 24 h. Cells were lysed and divided into three groups for immunoprecipitation: IgG negative control, MOV10 immunoprecipitation without RNase A treatment, and MOV10 immunoprecipitation with RNase A digestion after magnetic bead binding. Immunoprecipitants were analyzed by western blotting using Abs against MOV10, SADS-CoV N, and GAPDH. The density of SADS-CoV N protein bands relative to GAPDH was calculated, with the values of each negative control group normalized to 1. (**F–I**) Expression of MOV10 was upregulated following SADS-CoV infection. IPI-2I and ST cells were infected with SADS-CoV (MOI = 0.1) and harvested at 12 and 24 hpi. Cell lysates were analyzed by western blotting (**F and H**) using Abs against MOV10 and the SADS-CoV N protein. GAPDH was used as a protein loading control. The density of MOV10 bands relative to GAPDH was calculated, with the values of each negative control group normalized to 1. MOV10 mRNA levels were quantified by RT-qPCR (**G and I**). (**J**) Representative photomicrographs of viral antigen immunochemical staining in SADS-CoV-infected and uninfected jejunum and ileum tissues (bar: 200 µm). The virus in intestinal tissue cells was immunostained using mAb 3E9 against SADS-CoV N protein. (**K**) Viral RNA copy numbers in the jejunum and ileum samples were analyzed by RT-qPCR. (**L**) MOV10 levels in the jejunum and ileum of SADS-CoV-infected and uninfected piglets at 36 hpi were quantified by RT-qPCR. The mean and SD of the results from three independent experiments are shown (*P < 0.05).

The coronavirus N protein mediates the host innate immune response through multiple mechanisms, including modulation of host immune signaling pathways, interaction with host factors, and regulation of antiviral gene expression (26, 27). To identify host factors that inhibit SADS-CoV immune evasion, we performed immunoprecipitation with Ab 3E9 against the SADS-CoV N protein, followed by liquid chromatography‑tandem mass spectrometry (LC-MS/MS) analysis of the enriched samples (Fig. 1A). Among the identified host factors, MOV10 exhibited the highest fold change in protein expression (Fig. 1B). Co-immunoprecipitation (Co-IP) assays confirmed this interaction (Fig. 1C), and consistent results were further validated in Fig. S1A. Furthermore, to verify whether this interaction occurs under physiologically relevant infection conditions, we performed Co-IP assays using lysates from SADS-CoV-infected IPI-2I cells. As shown in Fig. 1D, endogenous MOV10 specifically co-immunoprecipitated with the viral N protein, confirming their interaction during SADS-CoV infection. Consistently, identical results were recapitulated in SADS-CoV-infected HEK293T cells (Fig. S1B). We then divided the N protein into three truncated regions to investigate the regions involved in the interaction (N1, N2, and N3; Fig. S1C). Our results demonstrated that MOV10 interacted with the N1 and N2 regions (Fig. S1D and E). To investigate whether the interaction between the SADS-CoV N protein and MOV10 was dependent on RNase activity, we subjected SADS-CoV-infected IPI-2I cell lysates to RNase A digestion followed by an endogenous Co-IP assay. We observed a reduction in the interaction between SADS-CoV N protein and MOV10 in the presence of RNase A, suggesting that the interaction between SADS-CoV N and MOV10 was likely RNA-dependent (Fig. 1E). In addition, we also overexpressed both SADS-CoV N protein and MOV10 in HEK293T cells with or without RNase A treatment. The Co-IP results recapitulated our earlier observations in SADS-CoV-infected IPI-2I cells, further suggesting that the binding between MOV10 and SADS-CoV N protein may rely on RNA intermediates (Fig. S1F).

To confirm the function of MOV10 in the replication of SADS-CoV, we first assessed expression of MOV10 *in vitro* and *in vivo* following SADS-CoV infection. IPI-2I and ST cells were infected with SADS-CoV (MOI = 0.1) for 12 and 24 h. We observed that both the protein (Fig. 1F and H) and mRNA (Fig. 1G and I) levels of MOV10 in SADS-CoV-infected cells showed an obvious increase compared to the control group. Three-day-old specific-pathogen-free (SPF) piglets were orally challenged with SADS-CoV, and unchallenged piglets served as mock controls. Jejunal and ileal tissues from both groups were harvested at 36 h post-infection (hpi), and successful infection was confirmed by immunohistochemistry (IHC) and reverse transcription quantitative real-time PCR (RT-qPCR) (Fig. 1J and K). Notably, the MOV10 mRNA levels were also significantly higher in the jejunum and ileum of SADS-CoV-infected piglets compared to controls (Fig. 1L). These findings revealed that SADS-CoV infection upregulated MOV10 expression *in vitro* and *in vivo*.

### MOV10 is a host restriction factor for SADS-CoV replication

**Fig 2:**
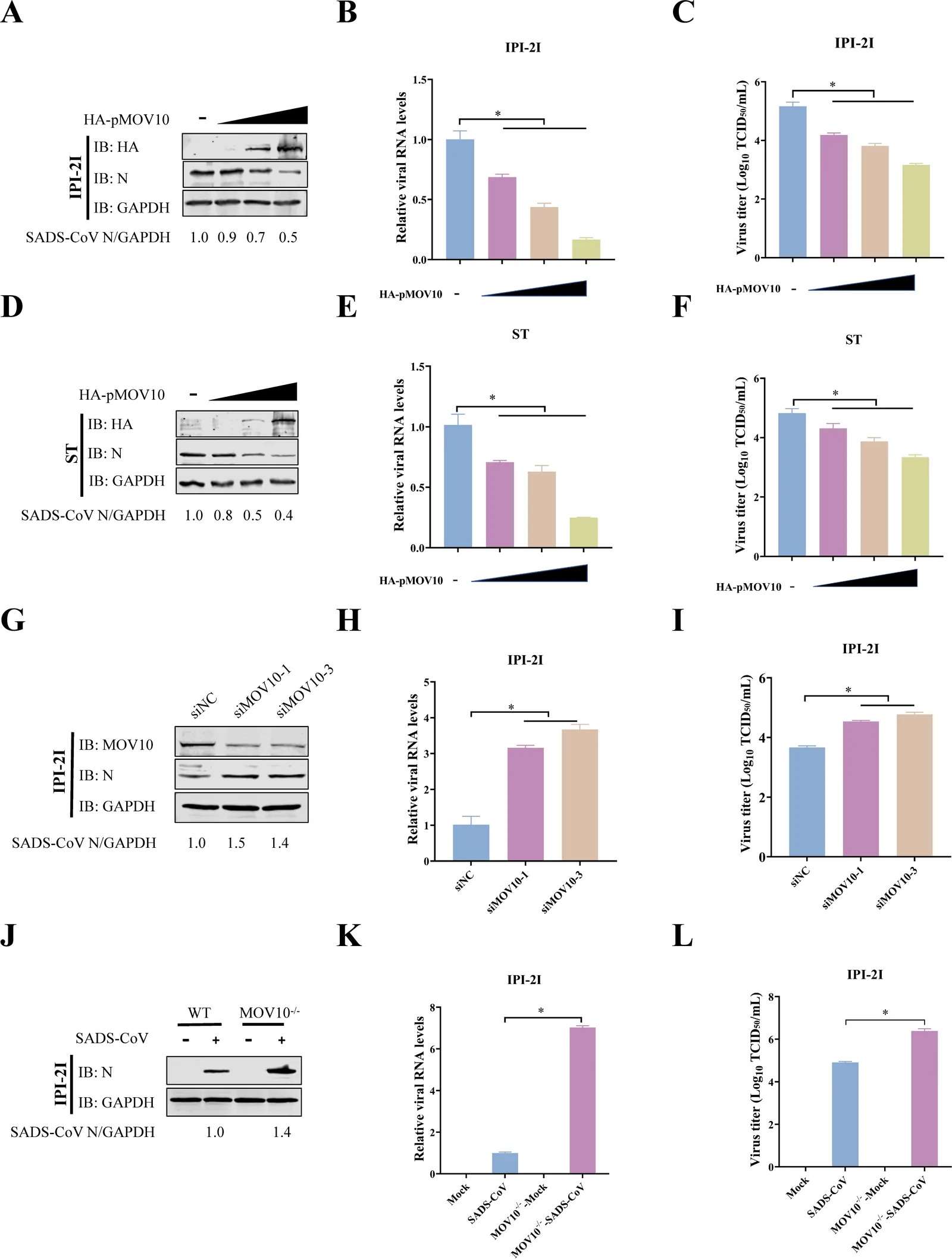
MOV10 is a host restriction factor for SADS-CoV infection. (**A–F**) Overexpression of MOV10 inhibited SADS-CoV replication. IPI-2I and ST cells were transfected with varying concentrations (0, 1, 3, and 5 μg) of full-length porcine MOV10 (HA-pMOV10) for 24 h, followed by infection with SADS-CoV (MOI = 0.1) for an additional 24 h. Cell lysates were analyzed by western blotting (**A and D**) using Abs against the HA tag, SADS-CoV N protein, and GAPDH. The density of the N protein band relative to GAPDH was calculated, with values from each negative control group normalized to 1. Viral RNA levels were quantified by RT-qPCR (**B and E**). Viral titers were determined using TCID_50_ assays (**C and F**). (**G–I**) Knockdown of MOV10 enhanced replication of SADS-CoV. IPI-2I cells were transfected with siMOV10-1, siMOV10-3, or negative control (siNC) at 50 nM for 48 h and then infected with SADS-CoV (MOI = 0.1) for 24 h. Lysates were analyzed by western blotting (**G**) using Abs against MOV10, SADS-CoV N protein, and GAPDH. Band density for SADS-CoV N/GAPDH was calculated, with values from the siNC group standardized to 1. Viral RNA levels were quantified by RT-qPCR (**H**). Viral titers were determined using TCID_50_ assays (**I**). (**J–L**) Knockout of MOV10 enhanced SADS-CoV replication. WT and MOV10^−/−^ IPI-2I cells were infected with SADS-CoV (MOI = 0.1) or uninfected for 24 h. Lysates were analyzed by western blotting (**J**). Density of SADS-CoV N bands relative to GAPDH was calculated using grayscale analysis. Viral RNA levels were quantified by RT-qPCR (**K**). Viral titers were determined using TCID_50_ assays (**L**). Mean and SD of the results from three independent experiments are shown (*P < 0.05).

To further elucidate the role of MOV10 in SADS-CoV replication, IPI-2I cells overexpressing porcine MOV10 were infected with SADS-CoV. We found that the levels of viral N protein in SADS-CoV-infected IPI-2I cells were significantly reduced (Fig. 2A and B). Additionally, a marked decrease in viral titer was also observed (Fig. 2C). We next extended our findings to ST cells. It is not surprising that SADS-CoV N protein expression (Fig. 2D), viral RNA levels (Fig. 2E), and viral titer (Fig. 2F) showed apparent decreases. Our findings demonstrated that overexpression of MOV10 suppressed the replication of SADS-CoV in a dose-dependent manner. Furthermore, three small interfering RNAs (siRNAs) specifically designed to target porcine MOV10 were employed to downregulate its expression in IPI-2I cells. Among these, siMOV10-1 and siMOV10-3 resulted in the most significant downregulation of MOV10 levels (Fig. S2A and B). We subsequently found that knockdown of MOV10 resulted in a pronounced increase in viral N protein and viral RNA levels (Fig. 2G and H) and enhanced the release of infectious virus (Fig. 2I). To further confirm MOV10 as a host restriction factor, we generated MOV10-deficient IPI-2I cells (MOV10^−/−^) through CRISPR-mediated genome editing (Fig. S3A through C). As shown in Fig. 2J through L, we observed significant upregulation of SADS-CoV N protein expression, viral RNA levels, and viral titers in MOV10^−/−^ cells compared with wild-type (WT) cells. Collectively, these results indicated that MOV10 is a host restriction factor for SADS-CoV replication.

### MOV10 inhibits SADS-CoV replication mainly through its N-terminal domain

**Fig 3:**
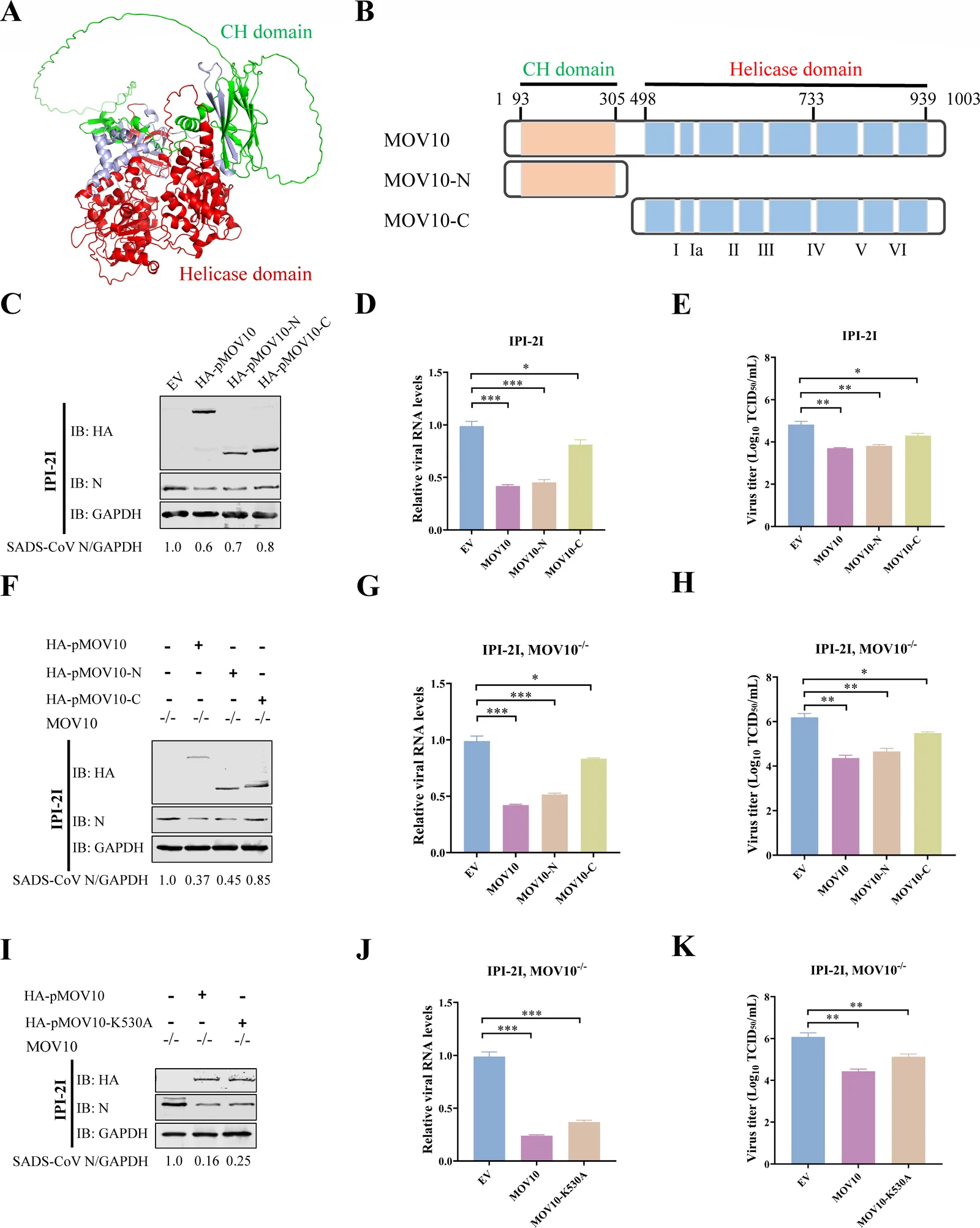
MOV10 suppressed SADS-CoV replication independent of its RNA helicase activity. (**A**) Structural of CH (green) and helicase (red) domains of porcine MOV10. (**B**) Schematic diagram of truncated MOV10 mutants. Numbers indicate amino acid positions. The orange boxes represent the CH domain. The blue boxes to the left and right represent helicase domains, respectively. The positions of the seven helicase motifs are indicated under the third bar. (**C–E**) The N-terminus of MOV10 was required for inhibition of SADS-CoV replication. IPI-2I cells were transfected with HA-pMOV10 or its truncations (HA-pMOV10-N and HA-pMOV10-C) for 24 h, followed by infection with SADS-CoV (MOI = 0.1) for an additional 24 h. Cell lysates were analyzed by western blotting (**C**). The density of SADS-CoV N bands relative to GAPDH was calculated using grayscale analysis. Viral RNA levels were quantified by RT-qPCR (**D**). Viral titers were determined using TCID_50_ assays (**E**). (**F–H**) Complementation of full-length MOV10, MOV10-N, and MOV10-C truncates restricts SADS-CoV replication in MOV10^⁻/⁻^ IPI-2I cells. MOV10^⁻/⁻^ IPI-2I cells were transfected with EV, HA-pMOV10, or its truncations (HA-pMOV10-N and HA-pMOV10-C), followed by SADS-CoV infection at an MOI of 0.1. Cell lysates were analyzed by western blotting (**F**). The density of SADS-CoV N bands relative to GAPDH was calculated using grayscale analysis. Viral RNA levels were quantified by RT-qPCR (**G**). Viral titers were determined using TCID_50_ assays (**H**). (**I–K**) Complementation of full-length MOV10 or helicase-dead MOV10-K530A mutant restricts SADS-CoV replication in MOV10^⁻/⁻^ IPI-2I cells. MOV10^⁻/⁻^ IPI-2I cells were transfected with EV, HA-pMOV10, or helicase site mutant (HA-pMOV10-K530A), followed by SADS-CoV infection at an MOI of 0.1. Cell lysates were analyzed by western blotting (**I**). The density of SADS-CoV N bands relative to GAPDH was calculated using grayscale analysis. Viral RNA levels were quantified by RT-qPCR (**J**). Viral titers were determined using TCID_50_ assays (**K**). The mean and SD of the results from three independent experiments are shown (*P < 0.05; **P < 0.01; ***P < 0.001).

MOV10 is an RNA helicase that contains an N-terminal CH-rich domain and a C-terminal helicase domain (Fig. 3A). The C-terminus of MOV10 (524–912 aa) contains seven helicase motifs (I, Ia, II, III, IV, V, and VI) required for its helicase activity, which have a DEAG fingerprint structure. Therefore, it is classified into the SF-1 family, and its N-terminus (93–305 aa) contains a Cys-His-rich (CH) domain (Fig. 3B) (28, 29). Wang et al. demonstrated that MOV10 inhibits Middle East respiratory syndrome coronavirus (MERS-CoV) through its helicase activity (30). To determine whether MOV10 suppresses SADS-CoV replication by a similar mechanism, we infected IPI-2I cells transfected with either an N-terminal or C-terminal plasmid with the virus. As shown in Fig. 3C and D, full-length MOV10 and its N-terminal domain significantly reduced the levels of N protein and viral RNA, whereas the C domain only resulted in a minor reduction. These findings were further confirmed by 50% tissue culture infective dose (TCID_50_) assays (Fig. 3E). Moreover, similar results were observed in ST cells, supporting the consistency of our observations (Fig. S1G through I). To validate our earlier findings from WT cell overexpression, we complemented MOV10^⁻/⁻^ IPI-2I cells with full-length MOV10, MOV10-N, or MOV10-C truncates before SADS-CoV infection. Consistent with prior data, full-length MOV10 and its N-terminal domain exerted stronger antiviral activity, while MOV10-C exhibited only weak inhibitory effects (Fig. 3F through H). We further verified the requirement of MOV10 helicase activity via the K530A helicase-dead mutant (18). We found that the K530A mutant retains potent antiviral activity, though its inhibitory effect is slightly weaker than that of full-length MOV10 (Fig. 3I through K). Interestingly, these results revealed that MOV10 primarily inhibits SADS-CoV replication through its N-terminal domain, rather than relying on its helicase activity. This finding suggested that, despite both viruses belonging to the same family of coronaviruses, the mechanism by which MOV10 inhibits SADS-CoV differs significantly from that of MERS-CoV.

### MOV10 positively regulates IFN-β response upon SADS-CoV infection

**Fig 4:**
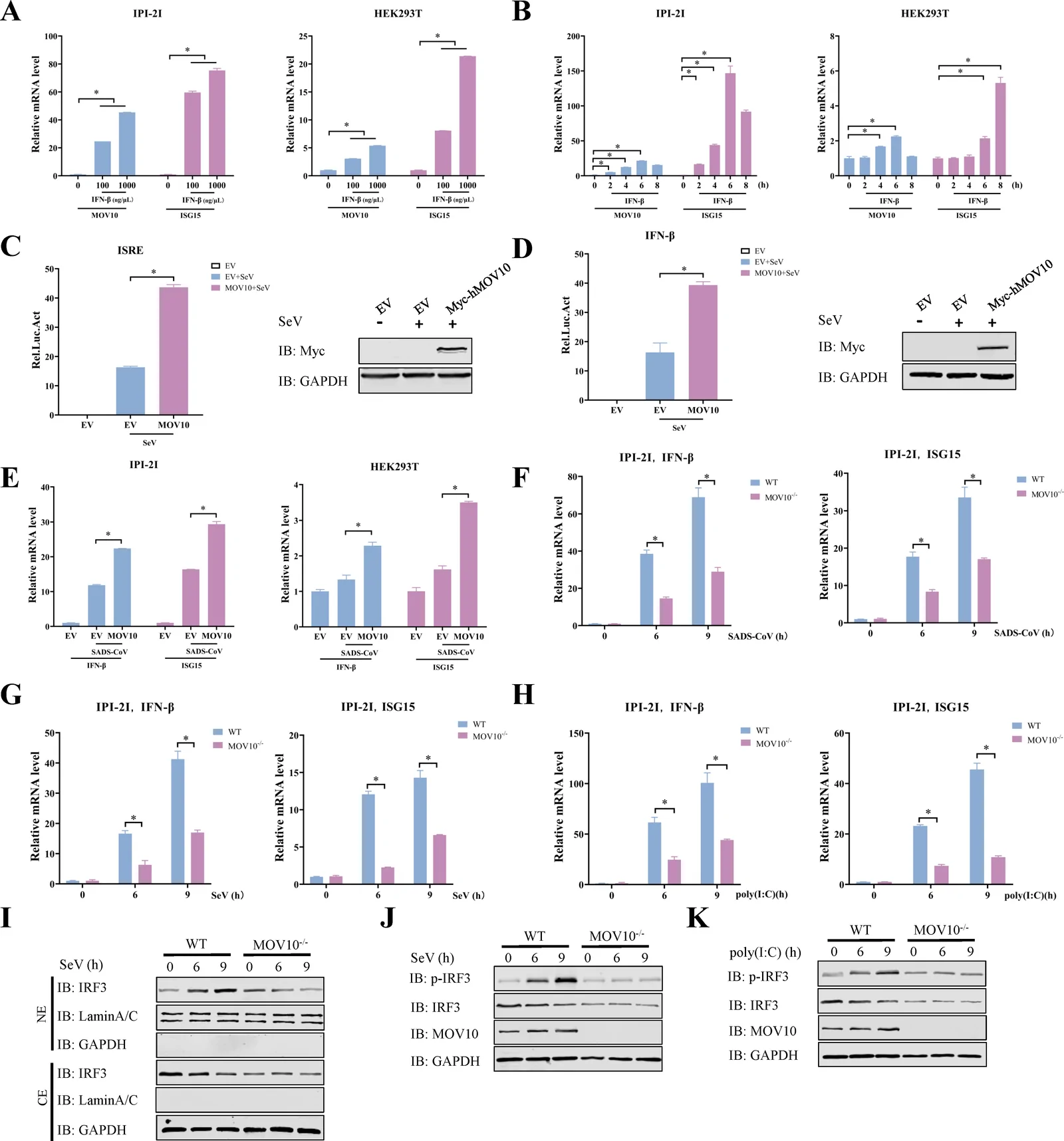
MOV10 positively regulated IFN-β response. (**A and B**) MOV10 was an ISG in IPI-2I and HEK293T cells. IPI-2I and HEK293T cells were treated with porcine or human recombinant IFN-β (0, 100, and 1,000 ng/µL) for 8 h. mRNA levels of ISG15 and MOV10 were then measured by RT-qPCR (**A**). IPI-2I and HEK293T cells were treated with recombinant IFN-β (100 ng/µL) for 0, 2, 4, 6, and 8 h, and mRNA levels of MOV10 and ISG15 were measured by RT-qPCR (**B**). (**C and D**) HEK293T cells were transfected with Myc-hMOV10 or empty vector (EV) for 24 h, followed by infection with SeV for 9 h. ISRE (**C**) or IFN-β (**D**) were detected by a luciferase reporter assay. Expression of MOV10 and GAPDH was determined by western blotting. (**E**) IPI-2I and HEK293T cells were transfected with Myc-hMOV10 or EV for 24 h and infected with SADS-CoV (MOI = 0.1) for an additional 24 h. mRNA levels of IFN-β and ISG15 were analyzed by RT-qPCR. (**F–H**) WT and MOV10^−/−^ IPI-2I cells were infected with SADS-CoV (**F**), SeV (**G**), or stimulated with 10 µg/mL poly(I:C) (**H**) for 6 and 9 h. mRNA levels of IFN-β and ISG15 were quantified by RT-qPCR. (**I**) WT and MOV10^−/−^ IPI-2I cells infected with SeV were harvested at 0, 6, and 9 h. Protein expression of IRF3, LaminA/C, and GAPDH in cytoplasmic (CE) and nuclear (NE) fractions was analyzed by western blotting. (**J and K**) WT and MOV10^−/−^ IPI-2I cells were infected with SeV or stimulated with 10 µg/mL poly(I:C) for 0, 6, and 9 h. Western blotting was performed using Abs against p-IRF3, IRF3, MOV10, and GAPDH. Mean and SD of the results from three independent experiments are shown (^*^P < 0.05).

Previous studies have indicated that MOV10 functions as an ISG (19, 31), but its role as an ISG in porcine intestinal cells, such as IPI-2I cells, remains unclear. We subsequently assessed expression of MOV10 and ISG15 in IPI-2I and HEK293T cells in response to stimulation with 100 and 1,000 ng/µL IFN-β. As expected, the levels of MOV10 and ISG15 were dramatically elevated in both cell lines treated with IFN-β compared to the control cells (Fig. 4A). In addition, both MOV10 and ISG15 reached their peak levels approximately 6–8 h after treatment with 100 ng/µL IFN-β (Fig. 4B). These results show that MOV10 is an ISG in IPI-2I and HEK293T cells. To further investigate the role of MOV10 in innate immunity, we analyzed its function by stimulation with RIG-I receptor ligands, such as Sendai virus (SeV) or poly(I:C) (32, 33). We first investigated the effect of MOV10 on IFN-sensitive response element (ISRE) or IFN-β expression following SeV infection. Results revealed that MOV10 markedly enhanced SeV-triggered activation of both the ISRE and IFN-β (Fig. 4C and D). We then found that MOV10 enhanced the levels of IFN genes, including IFN-β and ISG15, in IPI-2I and HEK293T cells infected with SADS-CoV (Fig. 4E).

Given that MOV10 positively regulated the IFN response, it was imperative to investigate whether knockout of MOV10 also affected this response. As shown in Fig. 4F through H, induction of IFN-β and ISG15 was markedly higher in WT IPI-2I cells than in MOV10^−/−^ cells following SADS-CoV infection, SeV infection, or poly(I:C) stimulation. The data suggest that MOV10 induced activation of the IFN-I pathway. To further validate these findings, we assessed the effect of MOV10 on phosphorylation of IRF3, a key marker of IFN-I pathway activation, following SeV infection or poly(I:C) treatment. We found that SeV-induced IRF3 nuclear translocation was dramatically suppressed in MOV10^−/−^ cells compared to WT cells (Fig. 4I). Consistently, MOV10 deficiency markedly impaired phosphorylation of IRF3 in response to SeV infection or poly(I:C) stimulation (Fig. 4J and K). Thus, these results demonstrate that MOV10 is important for host defense against viral infection by mediating IFN-β signaling activation.

### TRAF3 plays a crucial role in the MOV10-regulated innate immune pathway

**Fig 5:**
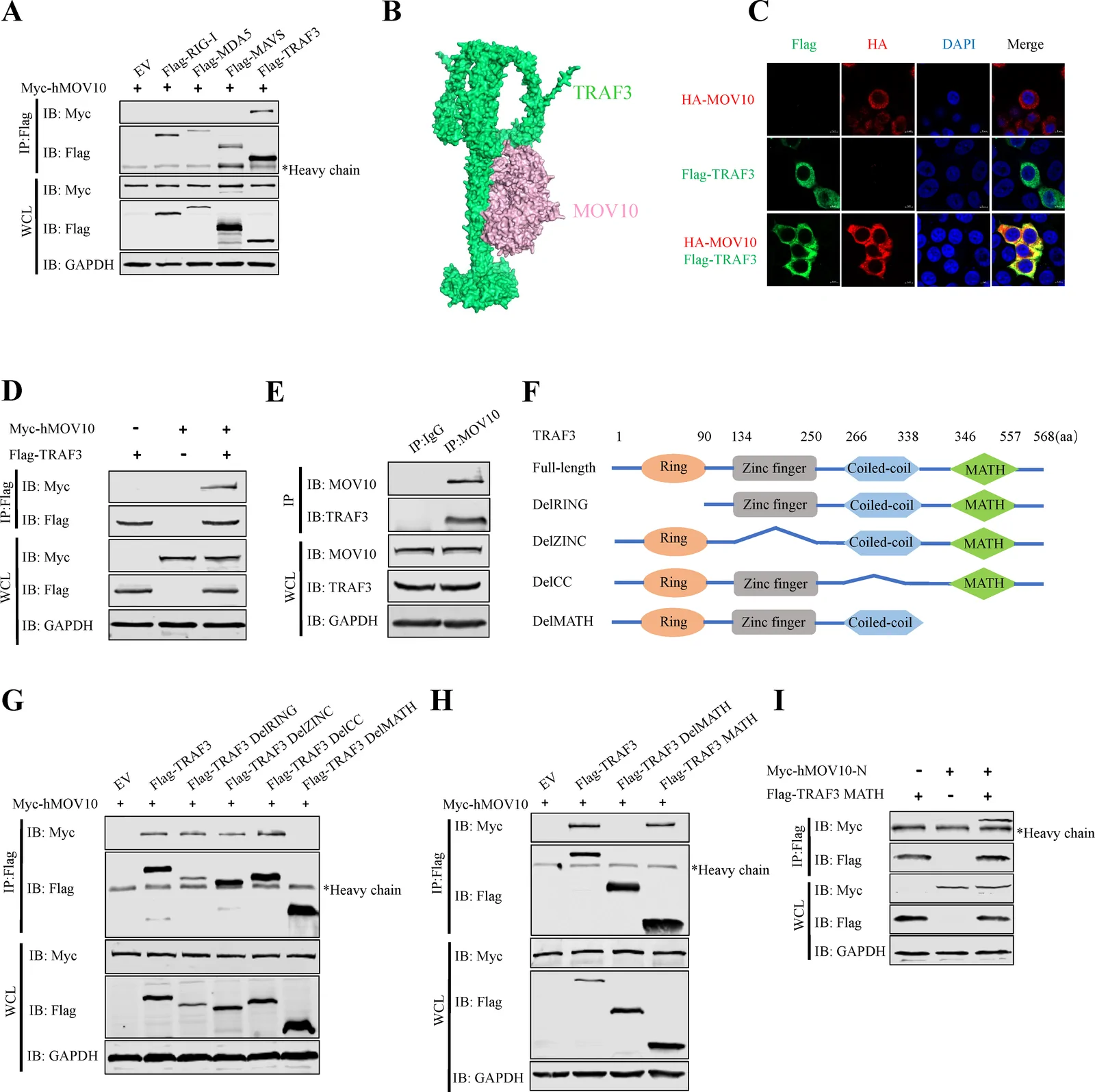
MOV10 interacted with TRAF3 in the RLR signaling pathway. (**A**) HEK293T cells were co-transfected with 2 μg of Myc-hMOV10 together with 2 μg of plasmid encoding Flag-tagged RIG-I, MDA5, MAVS, or TRAF3, respectively. Cell lysates were harvested and subjected to immunoprecipitation using Flag Ab. The immunoprecipitants were detected by western blotting using Abs against Myc, Flag, and GAPDH. (**B**) Cartoon representation of TRAF3 (green) bound to MOV10 (pink). (**C**) Co-localization of MOV10 and TRAF3. Vero E6 cells co-transfected with plasmids expressing HA-MOV10 (red) and Flag-TRAF3 (green). Merged images show co-localization of these proteins. Nuclei are highlighted by DAPI staining (blue) (bar: 5 µm). (**D**) Exogenous MOV10 interacted with TRAF3. HEK293T cells co-transfected with plasmids for Flag-TRAF3 and Myc-hMOV10, incubated for 24 h, and then collected for Co-IP assay using Flag Ab. (**E**) Endogenous MOV10 interacted with TRAF3. HEK293T cells were harvested for Co-IP using MOV10 Ab, with IgG Ab as the negative control. Immunoprecipitants were analyzed by western blotting using Abs against MOV10, TRAF3, and GAPDH. (**F**) Schematic of full-length TRAF3 and its serially truncated mutants. (**G and H**) Identification of the domain of TRAF3 required for interaction with MOV10. Plasmids encoding TRAF3, DelRING mutant, DelZINC mutant, DelCC mutant, and DelMATH mutant containing Flag tag were co-transfected into HEK293T cells with Myc-hMOV10, respectively. Lysates were harvested and subjected to immunoprecipitation using Flag Ab (**G**). Plasmids encoding TRAF3, DelMATH mutant, and MATH mutant containing Flag tag were co-transfected into HEK293T cells with Myc-hMOV10, respectively. Co-IP was performed using Flag Ab (**H**). (**I**) The N-terminus of MOV10 interacted with the MATH domain of TRAF3. HEK293T cells co-transfected with plasmids for Flag-TRAF3 MATH and Myc-hMOV10-N, incubated for 24 h, and then collected for Co-IP using Flag Ab.

RIG-I-mediated innate immune signaling is critical for host defense against RNA virus infection (34, 35). However, previous studies have reported that MOV10 protects against RNA virus infection by promoting the IFN-I response through an IKKε-mediated RNA sensing pathway, independent of the RIG-I–MAVS pathway (36). We were prompted to investigate its potential role against SADS-CoV by assessing its interaction with upstream regulators of IKKε, including RIG-I, MDA5, MAVS, and TRAF3. Notably, MOV10 was clearly associated with TRAF3, while no interaction was detected with RIG-I, MDA5, or MAVS (Fig. 5A). Interaction between MOV10 and TRAF3 was further predicted by AlphaFold3 (Fig. 5B). This interaction was confirmed by both confocal microscopy and Co-IP assays (Fig. 5C through E). These findings suggest that MOV10 plays a potential role in TRAF3-mediated innate immunity, further influencing downstream immune responses.

TRAF3 is a member of the tumor necrosis factor receptor associated factors and exhibits RING finger E3 ubiquitin ligase activity (37). Previous studies have demonstrated that TRAF3 promotes production of IFN-I by interacting with TBK1 and IKKε, playing a critical regulatory role in antiviral immune responses (38, 39). It is composed of RING finger domain (residues 68–77), zinc finger domain (residues 135–249), coiled-coil region domain (residues 267–338), and MATH domain (residues 415–560) (40). To identify the critical domain of TRAF3 involved in its interaction with MOV10, we constructed several TRAF3 deletion mutants (Fig. 5F). Their interactions were evaluated using Co-IP. We found that deletion of the C-terminal MATH domain of TRAF3 led to loss of interaction with MOV10 (Fig. 5G through I). Collectively, these results demonstrate that MOV10 interacts with TRAF3, and this binding is exclusively mediated by the MATH domain of TRAF3 and the N-terminal region of MOV10.

### MOV10 N-terminal domain enhances K63-linked polyubiquitination of TRAF3

**Fig 6:**
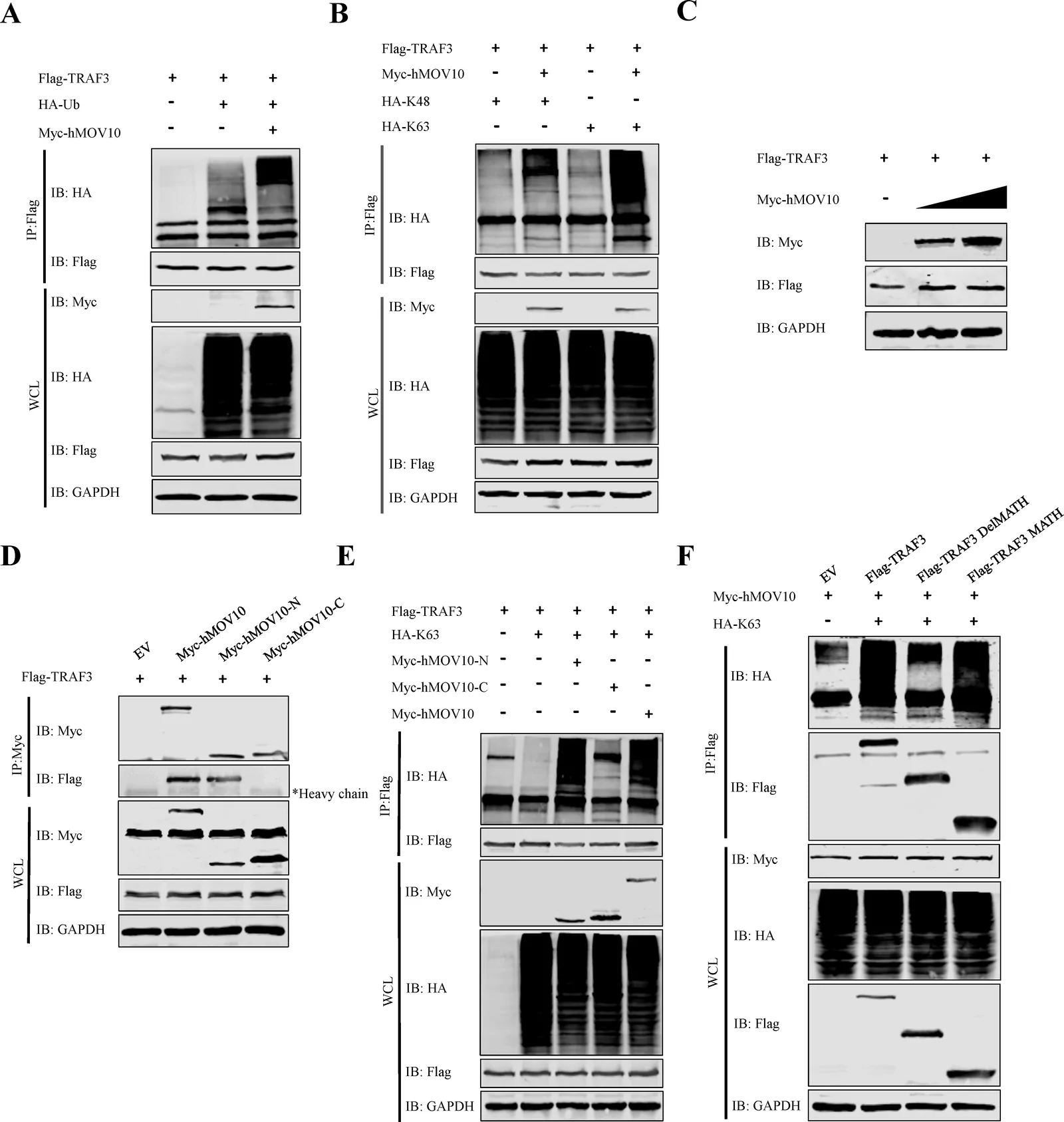
MOV10 enhanced K63-linked polyubiquitination of TRAF3. (**A**) HEK293T cells were transfected or co-transfected with plasmids encoding Flag-TRAF3, HA-ubiquitin (Ub), and Myc-hMOV10. Cell lysates were collected and subjected to immunoprecipitation using Flag Ab. (**B**) HEK293T cells were co-transfected with plasmids encoding Flag-TRAF3 and Myc-hMOV10, HA-ubiquitin (K48) or HA-ubiquitin (K63). Lysates were collected and subjected to immunoprecipitation using Flag Ab. (**C**) HEK293T cells were transfected with plasmids encoding Flag-TRAF3 and Myc-hMOV10 (0, 1, and 3 μg). Lysates were analyzed by western blotting. (**D**) HEK293T cells were co-transfected with plasmids encoding Flag-TRAF3, full-length Myc-hMOV10, or its mutants (Myc-hMOV10-N and Myc-hMOV10-C). Lysates were collected and subjected to immunoprecipitation using Myc Ab. (**E**) HEK293T cells were transfected or co-transfected with plasmids encoding Flag-TRAF3, HA-K63, Myc-hMOV10-N, Myc-hMOV10-C, and Myc-hMOV10. Lysates were collected and then subjected to immunoprecipitation using Flag Ab. (**F**) HEK293T cells were co-transfected with plasmids encoding Myc-hMOV10 and HA-K63, along with plasmids encoding Flag-TRAF3, Flag-TRAF3 DelMATH, or Flag-TRAF3 MATH. Lysates were collected and then subjected to immunoprecipitation using Flag Ab.

TRAF3 is a crucial signaling adaptor protein whose function is finely regulated by ubiquitination (41). To investigate whether MOV10 modulates ubiquitination of TRAF3, plasmids encoding MOV10, Ub, and TRAF3 were co-transfected into HEK293T cells. We observed that MOV10 enhanced the ubiquitination level of TRAF3 (Fig. 6A). Given that K63-linked ubiquitination of TRAF3 is required for induction of IFN-I (37, 41), we next examined the type of ubiquitin linkage in TRAF3 that was promoted by MOV10. After transfection with K48- or K63-specific ubiquitin linkage mutants, we found that MOV10 preferentially enhanced K63-linked, rather than K48-linked, polyubiquitination of TRAF3 (Fig. 6B). To further exclude the possibility that MOV10 played a role in regulating the K63-linked polyubiquitination of TRAF3, we examined the stability of TRAF3. Overexpression of increasing amounts of MOV10 in HEK293T cells did not significantly affect the protein level of TRAF3 (Fig. 6C). Considering that MOV10 suppressed SADS-CoV replication through the N-terminal domain; therefore, it was interesting to explore whether the N-terminal domain interacted with TRAF3 and enhanced its ubiquitination level. Results showed that the N-terminal domain of MOV10 was crucial for its interaction with TRAF3 (Fig. 6D). Moreover, the deletion mutant lacking the N-terminal domain failed to promote TRAF3 ubiquitination (Fig. 6E). Additionally, the MATH domain was essential for MOV10 to enhance the K63-linked polyubiquitination of TRAF3 (Fig. 6F). Collectively, these findings demonstrate that MOV10 enhances K63-linked polyubiquitination of TRAF3 via its N-terminal region, and the MATH domain of TRAF3 is indispensable for this MOV10-potentiated ubiquitination modification.

### TRIM24 mediates MOV10-enhanced K63-linked polyubiquitination of TRAF3

**Fig 7:**
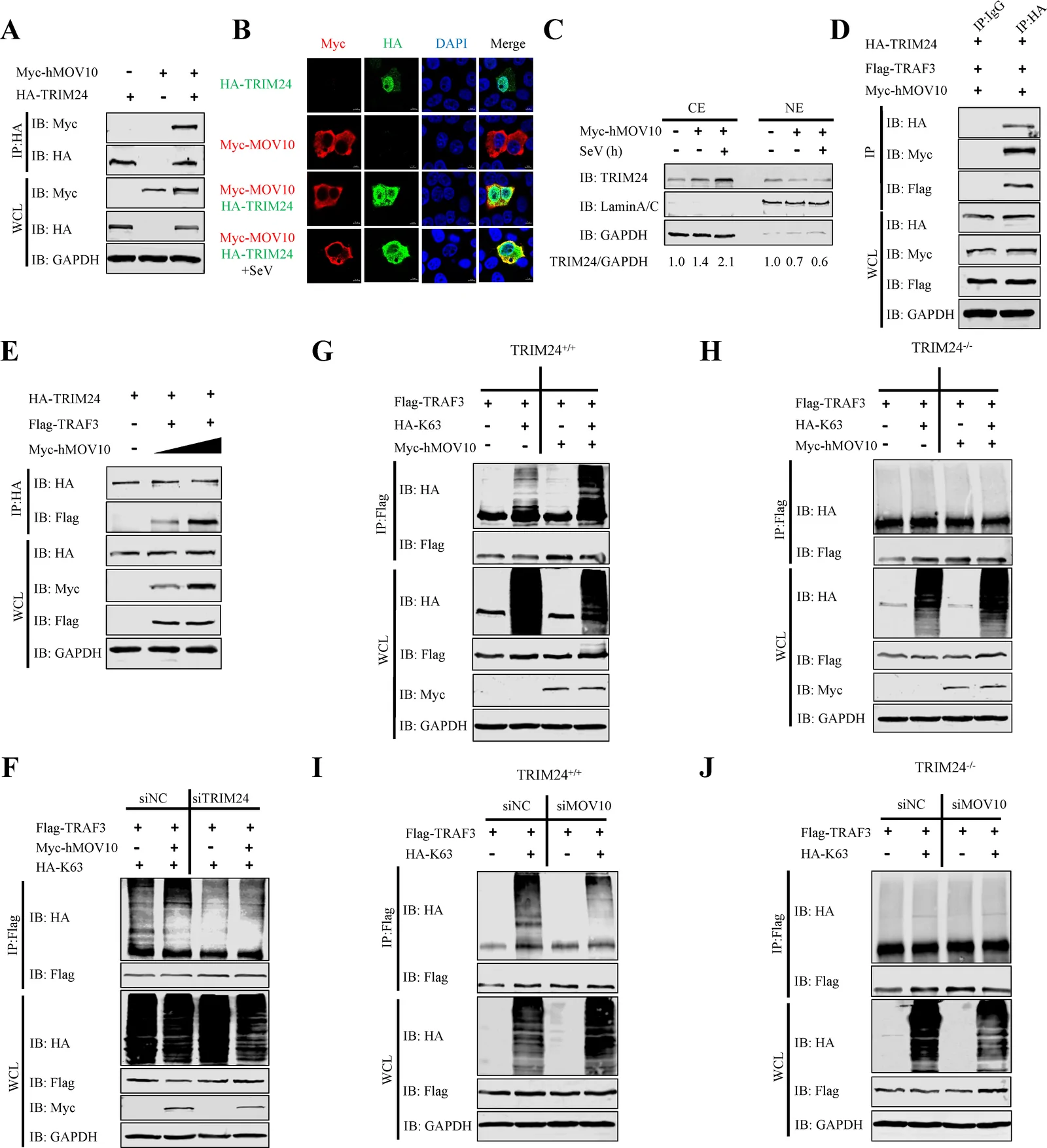
MOV10 promoted TRAF3 K63-linked polyubiquitination dependent on E3 ligase TRIM24. (**A**) HEK293T cells were co-transfected with Myc-hMOV10 and HA-TRIM24 for 24 h. Cell lysates were harvested and subjected to immunoprecipitation using HA Ab. (**B**) Co-localization of MOV10 and TRIM24. Plasmids expressing Myc-MOV10 (red) and HA-TRIM24 (green) were co-transfected into Vero E6 cells, which were then infected or not with SeV for 9 h. Merged images show co-localization of these proteins. Nuclei are highlighted by DAPI staining (blue) (bar: 5 µm). (**C**) HEK293T cells were transfected with plasmids expressing Myc-hMOV10 or EV and infected or not with SeV. Protein expression of TRIM24, LaminA/C, and GAPDH in cytoplasmic (CE) and nuclear (NE) fractions was assessed by western blotting. The density of TRIM24 bands relative to GAPDH was calculated using grayscale analysis. (**D**) HEK293T cells were co-transfected with plasmids expressing HA-TRIM24, Flag-TRAF3, and Myc-hMOV10. Cells were harvested for Co-IP using HA Ab, with IgG Ab as the negative control. (**E**) Plasmids encoding HA-TRIM24, Flag-TRAF3, or Myc-hMOV10 (0, 1, or 3 μg) were co-transfected into HEK293T cells. Cell lysates were collected and then subjected to immunoprecipitation using HA Ab. (**F**) HEK293T cells were transfected with either siTRIM24 or siNC (50 nM) for 36 h, followed by co-transfection with Flag-TRAF3, HA-K63, or Myc-hMOV10. Lysates were subjected to immunoprecipitation using Flag Ab. (**G and H**) WT (TRIM24^+/+^) and TRIM24 knockout (TRIM24^−/−^) HEK293T cells were co-transfected with Flag-TRAF3, HA-K63, or Myc-hMOV10. Lysates were subjected to immunoprecipitation using Flag Ab. (**I and J**) TRIM24^+/+^ and TRIM24^−/−^ HEK293T cells were transfected with either siMOV10 or siNC, followed by co-transfection with Flag-TRAF3 and HA-K63. Lysates were subjected to immunoprecipitation using Flag Ab.

Since MOV10 is not an E3 ligase, we hypothesized that MOV10 likely recruits an E3 ligase to promote K63-linked polyubiquitination of TRAF3. A recent study has shown that viral infection induces TRIM24 translocation from the nuclei to the cytoplasm and mediates K63-linked ubiquitination of TRAF3 (42). Therefore, we investigated whether the effect of MOV10 on the ubiquitination of TRAF3 was dependent on TRIM24. We first assessed the interaction between MOV10 and TRIM24. Co-IP revealed that MOV10 interacted with TRIM24 (Fig. 7A). To further validate this interaction under physiological conditions, we performed Co-IP assays using lysates from normal cells. As shown in Fig. S4A, endogenous MOV10 specifically co-immunoprecipitated with endogenous TRIM24. Confocal microscopy examined the co-localization of MOV10 and TRIM24 in Vero E6 cells. We observed that MOV10 was distributed throughout the cytoplasm, while TRIM24 was predominantly localized in the nuclei. Notably, co-transfection of MOV10 and TRIM24 led to a significant increase of TRIM24 in the cytoplasm, where it co-localized with MOV10. Zhu et al. previously demonstrated that SeV or vesicular stomatitis virus (VSV) challenge promotes nuclear export of TRAF3. Accordingly, we treated cells with combined SeV stimulation and MOV10 overexpression and found that the two treatments synergistically potentiate cytoplasmic redistribution of TRIM24 (Fig. 7B). Immunoblot analysis also indicated that MOV10 overexpression and SeV infection reduced nuclear TRIM24 levels while increasing its cytoplasmic levels (Fig. 7C).

To further confirm the function of MOV10 in promoting TRIM24-dependent TRAF3 K63-linked polyubiquitination, we focused on the interaction between MOV10, TRIM24, and TRAF3. As expected, we observed that MOV10, TRIM24, and TRAF3 form a complex through Co-IP assay (Fig. 7D). Interestingly, we also found that MOV10 enhanced the interaction between TRAF3 and TRIM24 in a dose-dependent manner (Fig. 7E). Subsequently, we investigated whether ubiquitination of TRAF3 induced by MOV10 occurred following TRIM24 knockdown. We selected siTRIM24-1 with the most significant knockdown effect for subsequent experiments (Fig. S2C and D). Compared to the control group, knockdown of TRIM24 in HEK293T cells inhibited the enhancement of K63-linked ubiquitination of TRAF3 by MOV10 (Fig. 7F). We then generated TRIM24 knockout (TRIM24^−/−^) HEK293T cells (Fig. S3D and E). As shown in Fig. 7G and H, overexpression of MOV10 in WT cells led to enhanced K63-linked polyubiquitination of TRAF3, which was not observed in TRIM24^−/−^ cells. These findings showed that TRIM24 is indispensable in the mediation of TRAF3 K63-linked polyubiquitination by MOV10. To further explore the relationship between MOV10 and TRIM24 in regulating TRAF3 ubiquitination, we knocked down MOV10 in the presence or absence of TRIM24. We observed that knockdown of MOV10 in the presence of TRIM24 reduced K63-linked polyubiquitination of TRAF3. However, knockdown of MOV10 in the absence of TRIM24 did not further decrease TRAF3 ubiquitination (Fig. 7I and J). In summary, these data suggest that MOV10 serves as a scaffold protein to recruit TRIM24 into the cytoplasm to promote TRAF3 ubiquitination to regulate anti-viral innate immunity.

### SADS-CoV N protein degrades TRAF3 through catalysis of K48-linked polyubiquitination

**Fig 8:**
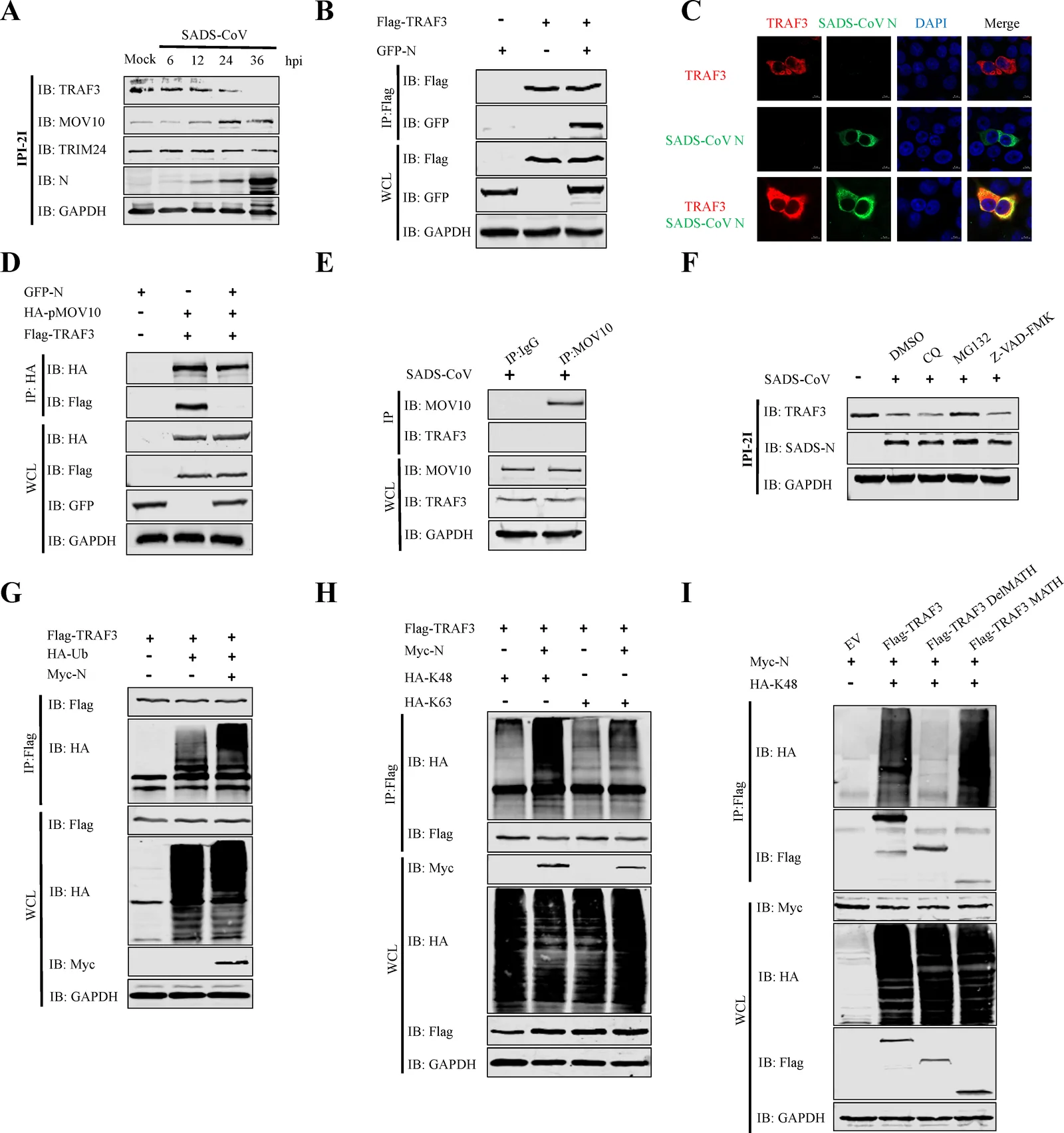
SADS-CoV N protein degraded TRAF3 by catalyzing K48-linked polyubiquitination. (**A**) IPI-2I cells were infected with SADS-CoV (MOI = 0.1). Cells were collected at 6, 12, 24, and 36 h and detected by western blotting using Abs against TRAF3, MOV10, TRIM24, SADS-CoV N protein, and GAPDH. (**B**) TRAF3 interacted with SADS-CoV N protein. HEK293T cells were co-transfected with Flag-TRAF3 and GFP-N. Cell extracts were subjected to immunoprecipitation using Flag Ab. (**C**) Co-localization of endogenous TRAF3 and SADS-CoV N protein. IPI-2I cells were infected with SADS-CoV (MOI = 0.1) for 24 h. Endogenous TRAF3 was stained red, and SADS-CoV N protein was stained green. Merged images show co-localization of these proteins. Nuclei are highlighted by DAPI staining (blue) (bar: 5 µm). (**D**) Plasmids encoding GFP-N, HA-pMOV10, and Flag-TRAF3 were transfected or co-transfected into HEK293T cells, respectively. Lysates were subjected to immunoprecipitation using HA Ab. (**E**) SADS-CoV infection disrupts the interaction between endogenous MOV10 and TRAF3. IPI-2I cells were infected with SADS-CoV (MOI = 0.1) for 24 h. Lysates were subjected to immunoprecipitation using MOV10 Ab, with IgG Ab as the negative control. Immunoprecipitants were analyzed by western blotting using Abs against MOV10, TRAF3, and GAPDH. (**F**) IPI-2I cells were infected with SADS-CoV (MOI = 0.1) and treated with CQ, MG132, Z-VAD-FMK, or DMSO (all 20 μM) for 5 h before sample collection. Western blotting was used to detect expression of TRAF3, SADS-CoV N protein, and GAPDH. (**G**) HEK293T cells were transfected or co-transfected with plasmids encoding Flag-TRAF3, HA-Ub, and Myc-N. Lysates were collected and then subjected to immunoprecipitation using Flag Ab. (**H**) HEK293T cells were co-transfected with plasmids encoding Flag-TRAF3, Myc-N, HA-K48, or HA-K63. Lysates were collected and then subjected to immunoprecipitation using Flag Ab. (**I**) HEK293T cells were co-transfected with Myc-N, HA-K48, Flag-TRAF3, or Flag-TRAF3 MATH. Lysates were subjected to immunoprecipitation using Flag Ab.

Our previous functional experiments have verified that MOV10 modulates K63-linked ubiquitination of TRAF3 to boost interferon production, yet sustained viral replication can still be observed at the late stage of SADS-CoV infection. Numerous RNA viruses have evolved diverse mechanisms to counteract IFN-mediated antiviral effects during late infection, thereby attenuating host-mediated restriction of viral replication (22). To further investigate whether the signaling pathway mediated by MOV10 underwent changes during the later stages of SADS-CoV infection, we measured expression of MOV10, TRAF3, and TRIM24 at different time points in IPI-2I cells infected with SADS-CoV. Notably, we observed that MOV10 expression increased progressively following viral infection, while TRIM24 levels remained unchanged. In contrast, expression of TRAF3 gradually decreased (Fig. 8A). As TRAF3 is a critical regulatory factor in the IFN-I signaling pathway, its reduced expression inhibits IFN production (39). The mechanism by which SADS-CoV interferes with TRAF3 function warrants further investigation. Li et al. demonstrated that the NS1 protein of influenza A virus impairs the interaction between MOV10 and viral nucleoprotein, which attenuates the antiviral activity of MOV10 (43). Furthermore, NS1 induces MOV10 degradation via the lysosomal pathway (43). Given that we have identified an interaction between the N protein of SADS-CoV and MOV10 and that expression of TRAF3 was downregulated during viral infection, we analyzed whether the N protein interacted with TRAF3, which was confirmed by Co-IP (Fig. 8B). Furthermore, we found that the MATH domain of TRAF3 was indispensable for its interaction with the N2 domain of SADS-CoV N protein (Fig. S4B through D). We also observed endogenous colocalization of TRAF3 and SADS-CoV N protein via confocal microscopy after SADS-CoV infection in IPI-2I cells (Fig. 8C). Consistently, obvious co-localization could also be detected upon exogenous overexpression of the two proteins (Fig. S4E). To further assess the relationship between the viral N protein, MOV10, and TRAF3, we then co-transfected the plasmids encoding these three proteins into HEK293T cells. Notably, Co-IP revealed a clear interaction between MOV10 and TRAF3, which was abolished in the presence of the N protein (Fig. 8D). Moreover, SADS-CoV infection also disrupted the interaction between endogenous MOV10 and TRAF3 (Fig. 8E). Consistently, exogenous overexpression of SADS-CoV N protein exhibited identical inhibitory effects on the endogenous interaction between MOV10 and TRAF3 (Fig. S4F).

Cellular protein degradation is primarily mediated by the ubiquitin-proteasome system and autophagy (44, 45). Additionally, proteolytic cleavage by caspases is a key feature of apoptosis, a form of programmed cell death (46). To investigate the pathways by which SADS-CoV degraded TRAF3, cells were treated with CQ (an autophagy inhibitor), MG132 (a ubiquitin-proteasome inhibitor), and Z-VAD-FMK (an apoptosis inhibitor) to measure TRAF3 expression. A cell viability assay showed that none of the three compounds exhibited cytotoxicity in IPI-2I cells at 20 μM (Fig. S4G). Compared to the DMSO group, TRAF3 expression showed no significant difference after CQ and Z-VAD-FMK treatment, while it was obviously upregulated after MG132 treatment (Fig. 8F). This suggested that the SADS-CoV N protein degraded TRAF3 via the ubiquitin-proteasome pathway. We next investigated whether the N protein modulated the ubiquitination level of TRAF3. Plasmids encoding SADS-CoV N protein, ubiquitin, and TRAF3 were co-transfected into HEK293T cells. As expected, a significant upregulation of TRAF3 ubiquitination levels was observed in the presence of SADS-CoV N protein (Fig. 8G). Given that different ubiquitin linkages are associated with distinctive functions, we next investigated the types of ubiquitin linkages in TRAF3 induced by the SADS-CoV N protein. Plasmids encoding K48- or K63-specific ubiquitin mutants were co-transfected with TRAF3 and the SADS-CoV N protein into HEK293T cells. We found that the N protein markedly enhanced K48-linked polyubiquitination of TRAF3 (Fig. 8H). Furthermore, the MATH domain of TRAF3 was essential for the SADS-CoV N protein-mediated K48-linked polyubiquitination of TRAF3 (Fig. 8I). Taken together, these results suggest that the SADS-CoV N protein antagonizes the host immune response via two distinct mechanisms: first, it disrupts the interaction between MOV10 and TRAF3, and second, it facilitates K48-linked polyubiquitination of TRAF3, thereby driving its degradation via the proteasome pathway.

## DISCUSSION

**Fig 9:**
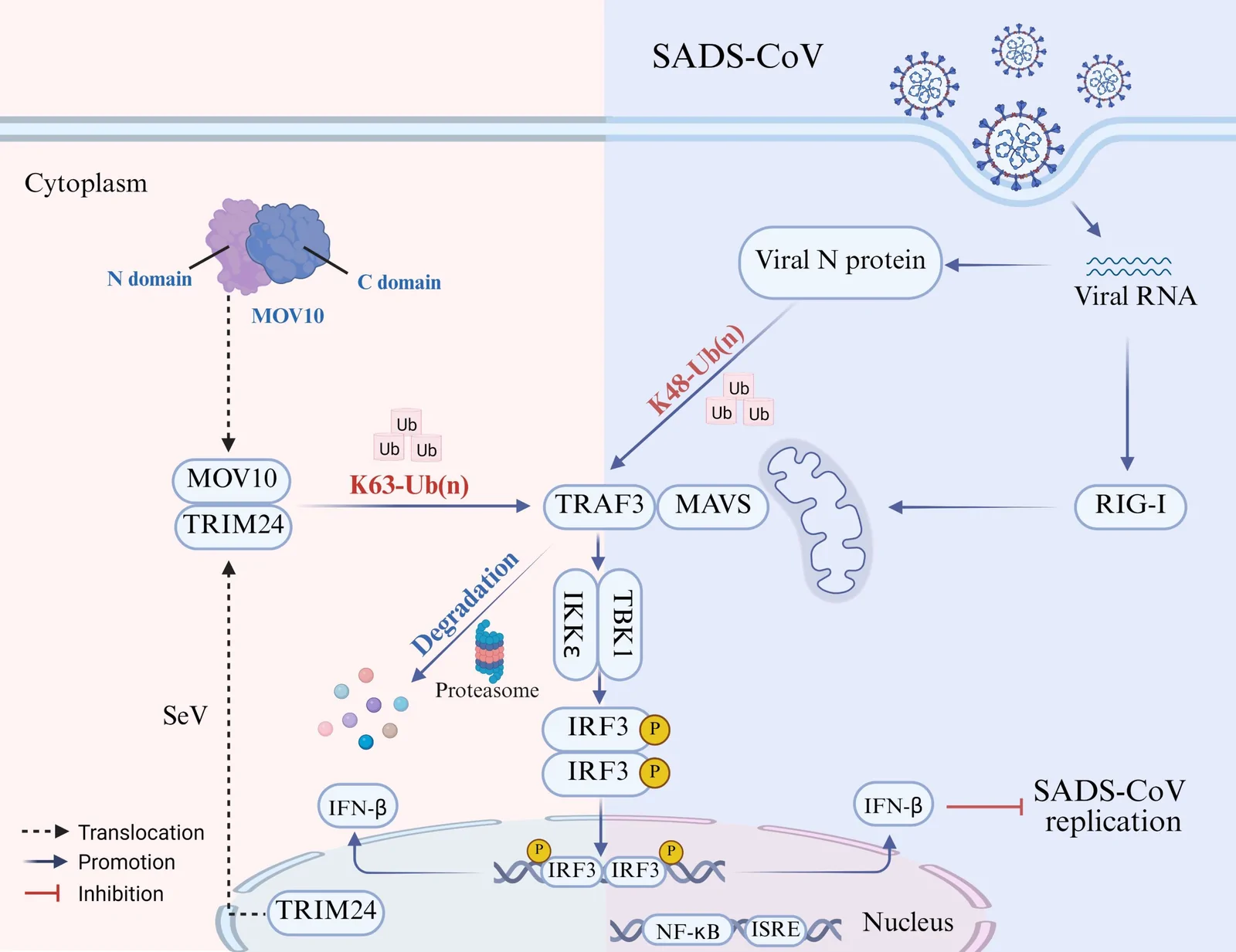
Models of MOV10 mechanisms in counteracting SADS-CoV and viral N protein antagonizing host innate immunity. To inhibit SADS-CoV replication, the host factor MOV10 enhanced the catalysis of K63-linked polyubiquitination of TRAF3 by E3 ubiquitin ligase TRIM24, thereby activating the RIG-I-mediated antiviral signaling pathway. On the other hand, the SADS-CoV N protein suppressed RIG-I-mediated antiviral signaling by promoting K48-linked polyubiquitination, leading to degradation of TRAF3.

In this study, we identified that MOV10, a superfamily 1 (SF1) RNA helicase, suppressed SADS-CoV replication by enhancing TRIM24-mediated K63-linked ubiquitination of TRAF3, which promotes IFN production. However, the antiviral activity of MOV10 was antagonized by the viral N protein, which disrupted the interaction of MOV10 with TRAF3. Furthermore, the viral N protein directly degraded TRAF3 through K48-linked ubiquitination, inhibiting IFN production during the later stages of SADS-CoV replication. These findings uncover the detailed antiviral mechanism of MOV10 and illustrate the specific immune escape strategy utilized by the SADS-CoV nucleocapsid protein to antagonize MOV10-dependent host innate immunity. (Fig. 9).

MOV10 has been reported to inhibit the replication of various viruses through distinct mechanisms. For instance, it suppresses influenza A virus replication by blocking the nuclear import of the viral nucleoprotein (43, 47). In bunyaviruses, MOV10 binds to the N-arm domain of the nucleoprotein, thereby preventing N polymerization and ribonucleoprotein (RNP) assembly (48). In HIV-1, MOV10 exerts antiviral effects by enhancing nuclear export of viral mRNA, interacting with the Gag protein, and being incorporated into viral particles, with its C-terminal region and helicase motifs being essential for this activity. Its interaction with Rev further facilitates nuclear export of viral RNA (49–51). Additionally, MOV10 restricts reverse transcription in viruses such as hepatitis B (52) virus and HIV (49). In the context of coronaviruses, MOV10 inhibits MERS-CoV and SARS-CoV-2 by interacting with the viral nucleocapsid protein and leveraging its helicase activity to suppress viral RNA replication (30, 53). MOV10 also exerts antiviral effects by promoting IFN production, as observed in VSV (36) and herpes simplex virus type 1 (54). In the present study, we demonstrated that MOV10 inhibited SADS-CoV replication by upregulating IFN-β expression, which was mediated by the N-terminal domain of MOV10. However, the molecular mechanism underlying the antiviral activity of MOV10 varies among different viruses. Within coronaviruses, the mechanism by which MOV10 inhibits SADS-CoV differs from that reported for MERS-CoV, where the helicase activity of MOV10 is required for its antiviral function (30). In contrast, our truncation mapping experiments demonstrated that the N-terminal domain of MOV10, rather than its C-terminal helicase domain, is responsible for inhibiting SADS-CoV replication and promoting TRAF3-mediated innate immune signaling. This domain-specific functional divergence is not limited to SADS-CoV and MERS-CoV. For instance, whether the helicase activity of MOV10 is necessary for its action against SARS-CoV-2 remains to be determined (53). Furthermore, extending beyond coronaviruses, MOV10’s anti-HIV-1 activity has been shown to require both N-terminal and C-terminal domains, with the N-terminal Gag-binding domain playing an essential role independent of helicase activity (28). Collectively, these differences suggest that MOV10 may exert broad antiviral effects through multiple mechanisms.

The production of IFN-I, initiated by recognition of pattern recognition receptors, plays a crucial role in the innate immune defense against viral infections. Our recent study showed that SADS-CoV infection activates the RIG-I and MAVS signaling pathways, while host protein DDX11 promotes activation of this signaling pathway through its interaction with RIG-I and MAVS. This activation cascade induces phosphorylation of TBK1 and IRF3, which subsequently induces IFN production (55). However, regarding the role of MOV10 in innate immunity, Cuevas et al. demonstrated that it restricts VSV proliferation by promoting IFN production via enhancement of IKKε, independently of TBK1 and the RIG-I-MAVS pathway (36). Furthermore, this conclusion is strengthened by the finding that MOV10 also inhibits HSV infection by promoting IFN production via an IKKε-mediated RNA-sensing pathway, independently of RIG-I, MDA-5, and MAVS (54). This suggests that MOV10 inhibits viral infection by inducing production of IFN through a unique mechanism. Here, we identified a novel role for MOV10 in TRAF3 ubiquitination, a mechanism not previously reported. Specifically, the N-terminal region of MOV10 enhanced K63-linked ubiquitination of TRAF3, with the MATH domain of TRAF3 playing a crucial role in this process. The function of TRAF3 in signaling is intricately linked to its ubiquitination status; a crucial post-translational modification recognized as a key regulator of host IFN-I signaling pathways (56, 57). TRAF3 undergoes two distinct types of ubiquitination: K48-linked polyubiquitination, which targets it for proteasomal degradation; and K63-linked polyubiquitination, which does not lead to degradation but likely modulates its function in signaling pathways (37, 41). Critically, K63-linked polyubiquitination of TRAF3 is essential for the recruitment and activation of TBK1/IKKε. This activation triggers the phosphorylation of the transcription factors IRF3/7, ultimately driving induction of IFN-I (42, 58). Therefore, by promoting K63-linked polyubiquitination of TRAF3, MOV10 facilitates IFN production, which in turn inhibits SADS-CoV replication.

MOV10 has not been reported to have E3 ubiquitin ligase activity. Therefore, it is likely that other E3 ligases are involved in ubiquitination of TRAF3, with MOV10 facilitating the process indirectly, possibly by interacting with these ligases or modifying their activity. Zhu et al. recently discovered that TRIM24 is a direct E3 ligase, which specifically induces K63-linked ubiquitination of TRAF3 during SeV/VSV infection (42). They found that RNA virus infection triggers CRM1-dependent TRIM24 translocation from the nuclei to the mitochondria, where it ubiquitinates TRAF3, activating the antiviral transcription factor IRF3, which then initiates IFN-I gene transcription. Consistent with these findings, we demonstrated that upon co-transfection of MOV10 and TRIM24, TRIM24 was also translocated from the nuclei to the cytoplasm, where it co-localized with MOV10. Furthermore, an interaction between MOV10, TRIM24, and TRAF3 was identified. Notably, our data indicated that TRIM24 promotes K63-linked polyubiquitination of TRAF3, with MOV10 enhancing the efficiency of this process. Our identification of the role of TRIM24 reinforces the critical importance of E3-ligase-mediated ubiquitination in controlling TRAF3 activity; a mechanism shared by several other enzymes including TRIM35, HECTD3, Nedd4l, RNF166, and RNF126, which have been identified as key regulators of immune signaling pathways (58–62). These enzymes regulate activation of IFN-I by mediating ubiquitination of TRAF3, thereby enhancing the host immune response against pathogens.

Viruses have evolved elaborate mechanisms to evade or inactivate the innate immune signaling pathway for their replication (63). Ubiquitin modifications and interplay between proteins in these signaling pathways enhance IFN-I expression. Conversely, viruses exploit the ubiquitin modification system to inhibit IFN-I production (64, 65). In addition to illustrating the anti-SADS-CoV activity and mechanism of MOV10, we also uncovered a viral counterstrategy mediated by the N protein of SADS-CoV. Mechanically, we found that the N protein inhibited the antiviral effect of MOV10 by disrupting the interaction between MOV10 and TRAF3. Moreover, the N protein promoted K48-linked polyubiquitination of TRAF3, leading to TRAF3 degradation via the ubiquitin-proteasome pathway. Through these two mechanisms, SADS-CoV effectively suppresses IFN production. Actually, the N protein of coronaviruses plays a key role in suppressing the secretion of host IFN-I, which facilitates viral evasion of the host immune surveillance system. Several viruses, including SARS-CoV-2 (11), SARS-CoV (12), PEDV (15), PDCoV (14), and MHV (13), inhibit IFN-β production through their N proteins using different mechanisms. Notably, Zhou et al. revealed that SADS-CoV antagonizes IFN-β production via blocking the interaction between TRAF3 and TBK1 (16). Building on this, our study further elucidated the molecular mechanism by which the SADS-CoV N protein promotes degradation of TRAF3 through the ubiquitin-proteasome pathway. This degradation likely impairs the ability of TRAF3 to interact with downstream signaling molecules, ultimately inhibiting production of IFN. Therefore, while Zhou et al. highlighted the disruption of TRAF3-TBK1 interaction as a key mechanism, our findings provide additional insights into how SADS-CoV N protein facilitates immune evasion by directly targeting TRAF3 for degradation.

How SADS-CoV N protein promotes K48-linked ubiquitination and degradation of TRAF3 remains to be fully elucidated. Based on our observation that N protein interacts with the MATH domain of TRAF3 and promotes its K48-linked ubiquitination and degradation, we propose two non-mutually exclusive models. First, N protein may function as an adaptor that recruits an unknown host E3 ligase to target TRAF3 for K48-linked ubiquitination, a strategy commonly employed by viral proteins to subvert host immunity. This speculation is consistent with the fact that many viral proteins hijack the host ubiquitin-proteasome system by serving as molecular bridges between viral targets and host E3 ligases (66, 67). Alternatively, although less common, N protein might possess intrinsic E3 ligase-like activity. Future biochemical studies using purified recombinant proteins, as well as co-immunoprecipitation coupled with mass spectrometry to identify N protein-interacting E3 ligases followed by functional validation, will be required to distinguish between these possibilities. Similarly, the mechanism by which N protein disrupts the MOV10-TRAF3 interaction requires further investigation. The observed disruption could result from N protein competing with MOV10 for TRAF3 binding, N protein inducing conformational changes in TRAF3 that preclude MOV10 binding, or N protein promoting degradation of MOV10 or TRAF3. Our preliminary data showing that N protein promotes TRAF3 degradation favor the latter possibility, but direct evidence is lacking. Future studies are needed to further explore the precise mechanism.

In summary, we have revealed a novel mechanism by which MOV10 antagonizes SADS-CoV. MOV10 suppresses SADS-CoV replication by promoting TRIM24-mediated K63-linked ubiquitination of TRAF3, which in turn enhances IFN production. Whether the MOV10-TRAF3 immune regulatory cascade identified in our study exerts broad antiviral effects against other enteric coronaviruses, including PEDV, TGEV, and PDCoV, remains to be further explored. Of note, our preliminary co-immunoprecipitation assays revealed that MOV10 interacts with the N proteins of PEDV, TGEV, and PDCoV, suggesting that MOV10 may broadly associate with N proteins from multiple enteric coronaviruses (data not shown). Based on this observation, we speculate that MOV10 may play a conserved role in promoting K63-linked ubiquitination of TRAF3 to activate innate immunity against diverse enteric coronaviruses. Furthermore, we also found that the N protein of SADS-CoV counters the antiviral effect of MOV10. The N protein not only inhibits the antiviral effect of MOV10 by disrupting the interaction between MOV10 and TRAF3 but also promotes K48-linked polyubiquitination of TRAF3, leading to TRAF3 degradation via the ubiquitin-proteasome pathway. These findings highlight the complex interplay between MOV10 and SADS-CoV, suggesting that MOV10 may function as a broad-spectrum antiviral agent. Further research is needed to fully understand its role across different viral infections. Collectively, these insights could inform the development of novel antiviral strategies, particularly those targeting SADS-CoV infection.

## MATERIALS AND METHODS

### Cells and viruses

Porcine intestinal epithelial cells (IPI-2I), swine testis cells (ST), African green monkey kidney cells (Vero E6), and human embryonic kidney cells (HEK293T) were kept in our laboratory and cultured in Dulbecco’s modified Eagle’s medium (DMEM; 11965092; Life Technologies, Carlsbad, CA, USA) supplemented with 100 mg/mL streptomycin, 100 units/mL penicillin, and 10% fetal bovine serum (10099141C; Gibco, Life Technologies). The cells were maintained at 37°C in a 5% CO_2_ incubator. SADS-CoV (GenBank accession number MF094681) was isolated from clinical samples with diarrheal symptoms from porcine herds in Guangdong Province of China and kept in our laboratory. SeV was purchased from the China Center for Type Culture Collection (Wuhan University, China) (10).

### Antibodies and reagents

SADS-CoV N protein-specific monoclonal antibody (mAb) 3E9 was prepared and maintained in our laboratory (68). Abs against Flag (ab205606), hemagglutinin (HA, ab9110), phosphorylated (p)-IRF3 (ab76493), IRF3 (ab68481), and GFP (ab290) were purchased from Abcam (Cambridge, MA, USA). Abs against Myc (M4439), HA (H9658), Flag (F1804), and glyceraldehyde 3-phosphate dehydrogenase (GAPDH, G9545) were purchased from Sigma-Aldrich (St. Louis, MO, USA). Abs against MOV10 (10370-1-AP), TRAF3 (18099-1-AP), TRIM24 (14208-1-AP), and HA (51064-2-AP) were purchased from Proteintech (Wuhan, China). Ab against Lamin A/C was purchased from Beyotime (Shanghai, China). IRDye 800CW goat anti-mouse IgG secondary Ab (926-32210) and IRDye 680RD goat anti-rabbit IgG secondary Ab (925-68071) were purchased from LiCor BioSciences (Lincoln, NE, USA). RNase A, Alexa Fluor 594 goat anti-rabbit IgG (H+L), Alexa Fluor 488 goat anti-mouse IgG (H+L), PageRuler Prestained Protein Ladder (Part No. 26616), and beads were purchased from Thermo Fisher Scientific (Waltham, MA, USA). Poly(I:C) was purchased from InvivoGen (Hong Kong, China) and was used at a final concentration of 10 µg/mL. Recombinant human IFN-β and swine IFN-β were purchased from R&D Systems (Minneapolis, MN, USA). MG132, Z-VAD-FMK, and CQ were purchased from MedChemExpress (Edison, NJ, USA). The Dual-Luciferase Reporter Assay System was purchased from Promega (Madison, WI, USA).

### Plasmids and transfection

Porcine full-length MOV10 (GenBank accession number XM_021090739.1) and human full-length MOV10 (GenBank accession number NM_001130079.3) were amplified from the cDNA of IPI-2I and HEK293T cells, respectively. The DNA fragment of MOV10 was inserted into the pCAGGS-HA (P0166; Miaoling Biology) or pCMV-Myc (635689; Clontech) vector via homologous recombination using the ClonExpress ultra one-step cloning kit (C116; Vazyme, Nanjing, China). The truncated mutants containing the porcine or human MOV10 N-terminal region (MOV10-N_93–305 aa) and C-terminal region (MOV10-C_524–912 aa) were cloned into the pCAGGS-HA or pCMV-Myc vector. The helicase-deficient point mutant of porcine MOV10 (K530A) was cloned into the pCAGGS-HA vector. SADS-CoV N protein (GenBank accession number ON911569.1) was cloned into the pAcGFP-C1 (632470; Clontech) and pCMV-Myc vector. The truncated mutants of SADS-CoV N (N1_1–146 aa, N2_147–249 aa, and N3_250–376 aa) were constructed on the basis of the full-length SADS-CoV N plasmid and inserted into the pAcGFP1-C1 vector, respectively. The ISRE and IFN-β promoter luciferase reporter plasmid, along with the internal reference plasmid (pRL-CMV), were stored in our laboratory as previously described (69). Human full-length TRAF3 (GenBank accession number NM_003300.4) was cloned into pCMV-Flag vector (635688; Clontech). The TRAF3 truncated mutants (DelRING_90–568 aa, DelZINC_1–134 aa and 250–568 aa, DelCC_1–266 aa and 338–568 aa, DelMATH_1–346 aa, and MATH_346–568 aa) were amplified from the full-length TRAF3 and inserted into the pCMV-Flag vector to generate the recombinant plasmids Flag-TRAF3-DelRING, Flag-TRAF3-DelZINC, Flag-TRAF3-DelCC, Flag-TRAF3-DelMATH, and Flag-TRAF3-MATH, respectively. The plasmids for expression of Flag-RIG-I, Flag-MDA5, and Flag-MAVS were preserved at our laboratory as previously described (55). The plasmids HA-ubiquitin (HA-Ub), HA-ubiquitin (K48), and HA-ubiquitin (K63) were stored in our laboratory. HEK293T and IPI-2I cells were transfected with the above plasmids using Lipofectamine 3000 transfection reagent (L3000001; Thermo Fisher Scientific).

### Screening of host factors associated with SADS-CoV N protein

Vero E6 cells were infected with SADS-CoV (MOI = 1) and incubated for 36 h. Cells were then collected and lysed. The resulting lysates were subjected to immunoprecipitation using Ab 3E9 targeting the N protein of SADS-CoV (1:100 dilution) (68), with IgG at the same concentration as a negative control. Enriched protein complexes were subsequently analyzed using LC-MS/MS to identify host factors potentially interacting with the viral N protein.

### Western blotting

Western blotting was performed as described previously (70). Cells were lysed on ice for 30 min using radio-immunoprecipitation assay buffer (R0278; Sigma-Aldrich, USA) supplemented with 1 mM phenylmethylsulfonyl fluoride solution (ST506-2; Beyotime). The lysate was then centrifuged at 12,000 × *g* for 5 min at 4°C to remove cellular debris. Sodium dodecyl sulfate polyacrylamide gel electrophoresis (SDS-PAGE) loading buffer (P0015L; Beyotime) was added to the supernatant and boiled for 10 min. Equal amounts of total protein were analyzed with 12.5% SDS-PAGE and transferred to nitrocellulose membranes (66485; Pall Corporation, Port Washington, NY, USA). After blocking with 5% skim milk for 1 h at room temperature (RT), the nitrocellulose membranes were incubated with primary Abs for 6–8 h at 4°C, followed by incubation with IRDye 800CW goat anti-mouse IgG (H+L) secondary Abs (1:10,000; 926-32210; LiCor BioSciences, USA) or IRDye 680RD goat anti-rabbit IgG (H+L) secondary Abs (1:10,000; 926-68071; LiCor BioSciences, USA) for 45 min in the dark. An Odyssey infrared imaging system (LiCor BioSciences, USA) was used to visualize the blots.

### Co-IP assay

Cells were lysed on ice for 30 min in IP lysis buffer (87788, Thermo Fisher Scientific) containing 1 mg/mL protease inhibitor cocktail (04693132001; Roche, Switzerland). The lysates were then incubated overnight at 4°C with primary Abs, followed by 6–8 h incubation with protein A/G magnetic beads at 4°C. After washing the bead-Ab-antigen complexes five times with lysis buffer, the immunoprecipitated proteins were collected for western blotting.

### RNA extraction and RT-qPCR

Total RNA was extracted from treated cells using the Simply P Total RNA Extraction Kit (BSC52S1; BioFlux, Brussels, Belgium) and reverse transcribed into cDNA using the PrimeScript IV 1st Strand cDNA Synthesis Mix (6215A; Takara, Japan). RT-qPCR (71) was conducted using the QuantStudio 5 Real-Time PCR System (Applied Biosystems, Foster City, CA, USA) with TB Green Premix Ex Taq II (RR820A; Takara, Japan). Relative quantitative mRNA levels were calculated using the 2^−∆∆Ct^ method. All experiments were performed at least three times. The sequences of the primers synthesized for RT-qPCR are listed in Table 1.

**TABLE 1:** Sense sequences are used for RT-qPCR in this study

Target	Primer	Sequence (5'−3')
SADS-CoV N	Forward	CCCCTAAACCGGCTCGTAA
	Reverse	CAGAATTAGGAACACGCTTCCA
hMOV10	Forward	AGAGTTTCCTGGTCGTTCGG
	Reverse	AGCTGATCTTGAAGTCGCGG
pMOV10	Forward	GGCCTACGTCACCAGGACTC
	Reverse	CATAAAAGATCTTGGCCAGCAGT
hGAPDH	Forward	CCCCTAAACCGGCTCGTAA
	Reverse	CAGAATTAGGAACACGCTTCCA
pGAPDH	Forward	CCCCTAAACCGGCTCGTAA
	Reverse	CAGAATTAGGAACACGCTTCCA
hIFN-β	Forward	GCAATTGAATGGAAGGCTTGA
	Reverse	CAGCGTCCTCCTTCTGGAACT
pIFN-β	Forward	AGCACTGGCTGGAATGAAACCG
	Reverse	CTCCAGGTCATCCATCTGCCCA
hISG15	Forward	ATCACCCAGAAGATCGGCG
	Reverse	TCGAAGGTCAGCCAGAACAG
pISG15	Forward	TGAAGATGCTGGGAGGCAAG
	Reverse	CACCCCATCCTGAAGCACAT

### Animal experiment and IHC assay

Six 3-day-old specific-pathogen-free (SPF) piglets were randomly assigned to either a challenge or control group. The challenge group was orally infected with 5 × 10^4^ TCID_50_ of SADS-CoV, whereas the control group was orally infected with the same volume of DMEM. We recorded the clinical symptoms (vomiting and diarrhea) and euthanized all piglets at 36 hpi. The euthanasia procedure followed the guidelines outlined in the aforementioned national standard. Piglets were first sedated via an intramuscular injection of xylazine (2.2 mg/kg body weight), followed by an intravenous overdose of pentobarbital sodium (150 mg/kg body weight) administered via the ear vein. Death was confirmed by the absence of corneal reflex and the cessation of both cardiac and respiratory activities. This method is consistent with the recommendations of the American Veterinary Medical Association (AVMA) Guidelines for the Euthanasia of Animals. Intestinal tissue samples were collected for RT-qPCR. Representative sections of the small intestine tissue samples (jejunum and ileum) were fixed in 4% paraformaldehyde and stored in 70% ethanol at 4°C. IHC was performed as previously described (70). Slides were incubated overnight at 4°C with mAb 3E9 (1:50) against the SADS-CoV N protein, followed by incubation with horseradish peroxidase-labeled goat anti-mouse IgG_1_ for 1 h. Immunocomplexes were detected using the 3,3ʹ-diaminobenzidine liquid substrate system.

### Construction of knockout cell lines

To generate MOV10^−/−^ IPI-2I cells and TRIM24^−/−^ HEK293T cells, the CRISPR/Cas9 gene-editing system was used. The single-guide RNA (sgRNA) was designed using the website (http://crispor.tefor.net/crispor.py). The specific sequences can be found in Table 2. The sgRNA-1 was cloned into the pX458-EGFP vector. The sgRNA-2 was cloned into the pX458-mCherry vector. Cells were transfected or co-transfected with pX458-sgRNA-1 and pX458-sgRNA-2. After cell culture, positive cells (exhibiting red and green dual fluorescence) were individually sorted into single clones on a 96-well plate using SH800S flow cytometer (Sony, Tokyo, Japan). After expanding and culturing the positive clones, cell samples were collected for sequencing and western blotting.

**TABLE 2:** Primer sequences of sgRNAs in this study

Target	Sequence (5'−3')
MOV10 sgRNA-1	TCGACCACTGGGCCGACGTG
MOV10 sgRNA-2	TGGAATGAAGATCGCCAATC
TRIM24 sgRNA-1	GTTGCATAAGTTTCATGCGA
TRIM24 sgRNA-2	GGCACTAGGTGAACGTATTG

### RNA interference

siRNAs targeting porcine MOV10 and human TRIM24 were designed and synthesized by Seven Biotechnology Co., Ltd. (Beijing, China). The siRNA target sequences used in the experiments are listed in Table 3. IPI-2I and HEK293T cells were seeded in 12-well plates, grown to 30%–40% confluency, and then transfected for 48 h with 50 nM siRNAs using Lipofectamine RNAiMAX Reagent (13778075; ThermoFisher Scientific). Following transfection, the cells were infected with SADS-CoV (MOI = 0.1), and samples were collected at 24 hpi.

**TABLE 3:** Primer sequences of siRNAs in this study

Target	Sense sequence (5'−3')	Antisense sequence (5'−3')
siMOV10-1	GCCUGGAUUUGAACCGCAA	UUGCGGUUCAAAUCCAGGC
siMOV10-2	GCAGGCCAUGAAGCACAUU	AAUGUGCUUCAUGGCCUGC
siMOV10-3	GCAGCUCGAUCUGGACUUU	AAAGUCCAGAUCGAGCUGC
siTRIM24-1	CACGAUUACUAGUGCAGCA	UGCUGCACUAGUAAUCGUG
siTRIM24-2	GAACGGUCCAGUCACCAAA	UUUGGUGACUGGACCGUUC
siTRIM24-3	CAGUUAGAGAGCCUUGCAA	UUGCAAGGCUCUCUAACUG

### Fifty percent tissue culture infective doses

Vero E6 cells were infected with 10-fold serial dilutions of each supernatant. At 4–6 days post-infection, cytopathic effects in cells were observed through microscopy. Viral titers were calculated using the Reed-Muench method.

### Dual-luciferase reporter assays

Dual-luciferase reporter assays were performed as described previously (55). HEK293T cells were seeded in 24-well plates. The cells were then transfected with either pCMV-Myc-MOV10 or an empty vector (EV), in addition to ISRE-Luc or IFN-β-Luc reporter constructs, along with an internal reference plasmid, pRL-CMV. After 24 h post-transfection (hpt), the cells were infected with SeV for 9 h. Following infection, the cells were lysed, and luciferase activity was assessed using a dual luciferase reporter assay kit (E1901; Promega, Madison, WI, USA).

### Immunofluorescence assay and confocal microscopy

Vero E6 cells cultured in 35-mm diameter dishes were transfected with specific plasmids. Cells were either infected or not infected with SeV after 24 hpt. The cells were fixed with precooled 4% paraformaldehyde (16005; Sigma-Aldrich) at RT for 30 min and permeabilized with 0.1% Triton X-100 (T8787; Sigma-Aldrich) at RT for 15 min. After blocking with 5% skim milk (P0216; Beyotime) at 4°C overnight, the cells were incubated with primary Abs for 1–4 h at RT. After three washes with PBST, the cells were incubated with corresponding fluorescein-conjugated secondary Abs (Alexa Fluor 594/488) at RT for 1 h in the dark. After PBST washing, cell nuclei were stained with 4’,6-diamidino-2-phenylindole (DAPI) for 15 min and then washed with PBST three times. Finally, the cells were directly observed using an LSM880-ZEISS confocal laser scanning microscope with Fast Airyscan (Zeiss, Germany).

### Cell viability assay

IPI-2I cells were grown in 96-well plates and then treated with drugs for 24 h. Cell viability was determined using Cell Counting Kit-8 (CCK-8; CK04, Dojindo Laboratories Co., Ltd., Kumamoto, Japan) according to the manufacturer’s instructions.

### Ubiquitination assay

To analyze the effect of MOV10 on ubiquitination of TRAF3 in HEK293T cells, HEK293T cells were co-transfected with the indicated plasmids, and then whole-cell lysates were immunoprecipitated with the corresponding Abs and analyzed by western blotting. To analyze the effect of SADS-CoV N protein on ubiquitination of TRAF3 in transfected cells, HEK293T cells were co-transfected with the indicated plasmids, and then whole-cell lysates were immunoprecipitated with the corresponding Abs and analyzed by western blotting.

### Statistical analysis

All results shown in the figures were presented, where appropriate, as mean and standard deviation (SD). The results of three independent experiments were analyzed with GraphPad Prism software (version 9.0; San Diego, CA, USA). Error bars represent SD. *P*-values are *P < 0.05; **P < 0.01; ***P < 0.001; ****P < 0.0001; ns, not significant.

## Data Availability

All relevant data are provided as figures within the paper and its supplemental material.

## SUPPLEMENTAL MATERIAL

Fig. S1 jvi.01215-26-s0001.tif Results of Co-IP assays.

Fig. S2 jvi.01215-26-s0002.tif Results of knockdown experiments.

Fig. S3 jvi.01215-26-s0003.tif Generation of MOV10^−/−^ cells through CRISPR-mediated genome editing.

Fig. S4 jvi.01215-26-s0004.tif Results of co-transfection assays.

Supplemental legends jvi.01215-26-s0005.docx Descriptive legends for Fig. S1 to S4.

ASM does not own the copyrights to Supplemental Material that may be linked to, or accessed through, an article. The authors have granted ASM a non-exclusive, world-wide license to publish the Supplemental Material files. Please contact the corresponding author directly for reuse.

## References

[1] Woo PCY, Lau SKP, Lam CSF, Lau CCY, Tsang AKL, Lau JHN, Bai R, Teng JLL, Tsang CCC, Wang M, Zheng B-J, Chan K-H, Yuen K-Y. 2012. Discovery of seven novel Mammalian and avian coronaviruses in the genus deltacoronavirus supports bat coronaviruses as the gene source of alphacoronavirus and betacoronavirus and avian coronaviruses as the gene source of gammacoronavirus and deltacoronavirus. J Virol 86:3995–4008. doi:10.1128/JVI.06540-11 PMC3302495 22278237

[2] Zhou P, Fan H, Lan T, Yang X-L, Shi W-F, Zhang W, Zhu Y, Zhang Y-W, Xie Q-M, Mani S, et al.. 2018. Fatal swine acute diarrhoea syndrome caused by an HKU2-related coronavirus of bat origin. Nature 556:255–258. doi:10.1038/s41586-018-0010-9 PMC7094983 29618817

[3] Pan Y, Tian X, Qin P, Wang B, Zhao P, Yang YL, Wang L, Wang D, Song Y, Zhang X, Huang YW. 2017. Discovery of a novel swine enteric alphacoronavirus (SeACoV) in southern China. Vet Microbiol 211:15–21. doi:10.1016/j.vetmic.2017.09.020 PMC7117260 29102111

[4] Gong L, Li J, Zhou Q, Xu Z, Chen L, Zhang Y, Xue C, Wen Z, Cao Y. 2017. A new bat-HKU2-like coronavirus in Swine, China, 2017. Emerg Infect Dis 23:1607–1609. doi:10.3201/eid2309.170915 PMC5572857 28654418

[5] Xu Z, Zhang Y, Gong L, Huang L, Lin Y, Qin J, Du Y, Zhou Q, Xue C, Cao Y. 2019. Isolation and characterization of a highly pathogenic strain of Porcine enteric alphacoronavirus causing watery diarrhoea and high mortality in newborn piglets. Transbound Emerg Dis 66:119–130. doi:10.1111/tbed.12992 PMC7168553 30103259

[6] Zhang T, Yao J, Yang Z, Wang J, Yang K, Yao L. 2024. Re-emergence of severe acute diarrhea syndrome coronavirus (SADS-CoV) in Henan, central China, 2023. Vet Microbiol 292:110049. doi:10.1016/j.vetmic.2024.110049 38493699

[7] Le NP, Le BT, Le VP, Park JE. 2025. Molecular characterization of swine acute diarrhea syndrome coronavirus detected in Vietnamese pigs. Vet Res 56:4. doi:10.1186/s13567-024-01445-0 PMC11720870 39789654

[8] Chen CJ, Sugiyama K, Kubo H, Huang C, Makino S. 2004. Murine coronavirus nonstructural protein p28 arrests cell cycle in G0/G1 phase. J Virol 78:10410–10419. doi:10.1128/JVI.78.19.10410-10419.2004 PMC516409 15367607

[9] Hurst KR, Ye R, Goebel SJ, Jayaraman P, Masters PS. 2010. An interaction between the nucleocapsid protein and a component of the replicase-transcriptase complex is crucial for the infectivity of coronavirus genomic RNA. J Virol 84:10276–10288. doi:10.1128/JVI.01287-10 PMC2937748 20660183

[10] Zhang J, Shi H, Zhang L, Feng T, Chen J, Zhang X, Ji Z, Jing Z, Zhu X, Liu D, Yang X, Zeng M, Shi D, Feng L. 2024. Swine acute diarrhea syndrome coronavirus nucleocapsid protein antagonizes the IFN response through inhibiting TRIM25 oligomerization and functional activation of RIG-I/TRIM25. Vet Res 55:44. doi:10.1186/s13567-024-01303-z PMC11000385 38589930

[11] Mu J, Fang Y, Yang Q, Shu T, Wang A, Huang M, Jin L, Deng F, Qiu Y, Zhou X. 2020. SARS-CoV-2 N protein antagonizes type I interferon signaling by suppressing phosphorylation and nuclear translocation of STAT1 and STAT2. Cell Discov 6:65. doi:10.1038/s41421-020-00208-3 PMC7490572 32953130

[12] Hu Y, Li W, Gao T, Cui Y, Jin Y, Li P, Ma Q, Liu X, Cao C. 2017. The severe acute respiratory syndrome coronavirus nucleocapsid inhibits type I interferon production by interfering with TRIM25-mediated RIG-I ubiquitination. J Virol 91:e00834–17. doi:10.1128/JVI.02143-16 PMC5375661 28148787

[13] Ding Z, Fang L, Yuan S, Zhao L, Wang X, Long S, Wang M, Wang D, Foda MF, Xiao S. 2017. The nucleocapsid proteins of mouse hepatitis virus and severe acute respiratory syndrome coronavirus share the same IFN-β antagonizing mechanism: attenuation of PACT-mediated RIG-I/ MDA5 activation. Oncotarget 8:49655–49670. doi:10.18632/oncotarget.17912 PMC5564796 28591694

[14] Likai J, Shasha L, Wenxian Z, Jingjiao M, Jianhe S, Hengan W, Yaxian Y. 2019. Porcine deltacoronavirus nucleocapsid protein suppressed IFN-β production by interfering porcine RIG-I dsRNA-binding and K63-linked polyubiquitination. Front Immunol 10:1024. doi:10.3389/fimmu.2019.01024 PMC6521028 31143181

[15] Ding Z, Fang L, Jing H, Zeng S, Wang D, Liu L, Zhang H, Luo R, Chen H, Xiao S. 2014. Porcine epidemic diarrhea virus nucleocapsid protein antagonizes beta interferon production by sequestering the interaction between IRF3 and TBK1. J Virol 88:8936–8945. doi:10.1128/JVI.00700-14 PMC4136253 24872591

[16] Zhou Z, Sun Y, Xu J, Tang X, Zhou L, Li Q, Lan T, Ma J. 2021. Swine acute diarrhea syndrome coronavirus nucleocapsid protein antagonizes interferon-β production via blocking the interaction between TRAF3 and TBK1. Front Immunol 12:573078. doi:10.3389/fimmu.2021.573078 PMC7937869 33692778

[17] Meister G, Landthaler M, Peters L, Chen PY, Urlaub H, Lührmann R, Tuschl T. 2005. Identification of novel argonaute-associated proteins. Curr Biol 15:2149–2155. doi:10.1016/j.cub.2005.10.048 16289642

[18] Gregersen LH, Schueler M, Munschauer M, Mastrobuoni G, Chen W, Kempa S, Dieterich C, Landthaler M. 2014. MOV10 Is a 5′ to 3′ RNA helicase contributing to UPF1 mRNA target degradation by translocation along 3′ UTRs. Mol Cell 54:573–585. doi:10.1016/j.molcel.2014.03.017 24726324

[19] Schoggins JW, Wilson SJ, Panis M, Murphy MY, Jones CT, Bieniasz P, Rice CM. 2011. A diverse range of gene products are effectors of the type I interferon antiviral response. Nature 472:481–485. doi:10.1038/nature09907 PMC3409588 21478870

[20] Shaw AE, Hughes J, Gu Q, Behdenna A, Singer JB, Dennis T, Orton RJ, Varela M, Gifford RJ, Wilson SJ, Palmarini M. 2017. Fundamental properties of the mammalian innate immune system revealed by multispecies comparison of type I interferon responses. PLoS Biol 15:e2004086. doi:10.1371/journal.pbio.2004086 PMC5747502 29253856

[21] Rehwinkel J, Gack MU. 2020. RIG-I-like receptors: their regulation and roles in RNA sensing. Nat Rev Immunol 20:537–551. doi:10.1038/s41577-020-0288-3 PMC7094958 32203325

[22] García-Sastre A. 2017. Ten strategies of interferon evasion by viruses. Cell Host Microbe 22:176–184. doi:10.1016/j.chom.2017.07.012 PMC5576560 28799903

[23] Khan R, Khan A, Ali A, Idrees M. 2019. The interplay between viruses and TRIM family proteins. Rev Med Virol 29:e2028. doi:10.1002/rmv.2028 30609250

[24] Castanier C, Zemirli N, Portier A, Garcin D, Bidère N, Vazquez A, Arnoult D. 2012. MAVS ubiquitination by the E3 ligase TRIM25 and degradation by the proteasome is involved in type I interferon production after activation of the antiviral RIG-I-like receptors. BMC Biol 10:44. doi:10.1186/1741-7007-10-44 PMC3372421 22626058

[25] Yan J, Li Q, Mao AP, Hu MM, Shu HB. 2014. TRIM4 modulates type I interferon induction and cellular antiviral response by targeting RIG-I for K63-linked ubiquitination. J Mol Cell Biol 6:154–163. doi:10.1093/jmcb/mju005 24755855

[26] Zhang S, Wang L, Cheng G. 2022. The battle between host and SARS-CoV-2: Innate immunity and viral evasion strategies. Molecular Therapy 30:1869–1884. doi:10.1016/j.ymthe.2022.02.014 PMC8842579 35176485

[27] Yao T, Foo C, Zheng G, Huang R, Li Q, Shen J, Wang Z. 2023. Insight into the mechanisms of coronaviruses evading host innate immunity. Biochim Biophys Acta Mol Basis Dis 1869:166671. doi:10.1016/j.bbadis.2023.166671 PMC9968664 36858323

[28] Abudu A, Wang X, Dang Y, Zhou T, Xiang SH, Zheng YH. 2012. Identification of molecular determinants from Moloney leukemia virus 10 homolog (MOV10) protein for virion packaging and anti-HIV-1 activity. J Biol Chem 287:1220–1228. doi:10.1074/jbc.M111.309831 PMC3256871 22105071

[29] Li X, Zhang J, Jia R, Cheng V, Xu X, Qiao W, Guo F, Liang C, Cen S. 2013. The MOV10 helicase inhibits LINE-1 mobility. J Biol Chem 288:21148–21160. doi:10.1074/jbc.M113.465856 PMC3774381 23754279

[30] Wang L, Sola I, Enjuanes L, Zuñiga S. 2021. MOV10 helicase interacts with coronavirus nucleocapsid protein and has antiviral activity. mBio 12:e0131621. doi:10.1128/mBio.01316-21 PMC8546642 34517762

[31] Hertzog P, Forster S, Samarajiwa S. 2011. Systems biology of interferon responses. J Interferon Cytokine Res 31:5–11. doi:10.1089/jir.2010.0126 21226606

[32] Kato H, Takeuchi O, Sato S, Yoneyama M, Yamamoto M, Matsui K, Uematsu S, Jung A, Kawai T, Ishii KJ, Yamaguchi O, Otsu K, Tsujimura T, Koh C-S, Reis e Sousa C, Matsuura Y, Fujita T, Akira S. 2006. Differential roles of MDA5 and RIG-I helicases in the recognition of RNA viruses. Nature 441:101–105. doi:10.1038/nature04734 16625202

[33] Yoneyama M, Kikuchi M, Natsukawa T, Shinobu N, Imaizumi T, Miyagishi M, Taira K, Akira S, Fujita T. 2004. The RNA helicase RIG-I has an essential function in double-stranded RNA-induced innate antiviral responses. Nat Immunol 5:730–737. doi:10.1038/ni1087 15208624

[34] Kawai T, Akira S. 2006. Innate immune recognition of viral infection. Nat Immunol 7:131–137. doi:10.1038/ni1303 16424890

[35] Thompson MR, Kaminski JJ, Kurt-Jones EA, Fitzgerald KA. 2011. Pattern recognition receptors and the innate immune response to viral infection. Viruses 3:920–940. doi:10.3390/v3060920 PMC3186011 21994762

[36] Cuevas RA, Ghosh A, Wallerath C, Hornung V, Coyne CB, Sarkar SN. 2016. MOV10 provides antiviral activity against RNA viruses by enhancing RIG-I-MAVS-independent IFN induction. J Immunol 196:3877–3886. doi:10.4049/jimmunol.1501359 PMC4868630 27016603

[37] Lin WW, Hostager BS, Bishop GA. 2015. TRAF3, ubiquitination, and B-lymphocyte regulation. Immunol Rev 266:46–55. doi:10.1111/imr.12299 PMC4473794 26085206

[38] Oganesyan G, Saha SK, Guo B, He JQ, Shahangian A, Zarnegar B, Perry A, Cheng G. 2006. Critical role of TRAF3 in the toll-like receptor-dependent and -independent antiviral response. Nature 439:208–211. doi:10.1038/nature04374 16306936

[39] Häcker H, Redecke V, Blagoev B, Kratchmarova I, Hsu L-C, Wang GG, Kamps MP, Raz E, Wagner H, Häcker G, Mann M, Karin M. 2006. Specificity in toll-like receptor signalling through distinct effector functions of TRAF3 and TRAF6. Nature 439:204–207. doi:10.1038/nature04369 16306937

[40] Zhu K, Wang X, Ju LG, Zhu Y, Yao J, Wang Y, Wu M, Li LY. 2015. WDR82 negatively regulates cellular antiviral response by mediating TRAF3 polyubiquitination in multiple cell lines. J Immunol 195:5358–5366. doi:10.4049/jimmunol.1500339 PMC4654227 26519536

[41] Tseng P-H, Matsuzawa A, Zhang W, Mino T, Vignali DAA, Karin M. 2010. Different modes of ubiquitination of the adaptor TRAF3 selectively activate the expression of type I interferons and proinflammatory cytokines. Nat Immunol 11:70–75. doi:10.1038/ni.1819 PMC2872790 19898473

[42] Zhu Q, Yu T, Gan S, Wang Y, Pei Y, Zhao Q, Pei S, Hao S, Yuan J, Xu J, Hou F, Wu X, Peng C, Wu P, Qin J, Xiao Y. 2020. TRIM24 facilitates antiviral immunity through mediating K63-linked TRAF3 ubiquitination. J Exp Med 217:e20192033. doi:10.1084/jem.20192083 PMC7336305 32324863

[43] Li J, Hu S, Xu F, Mei S, Liu X, Yin L, Zhao F, Zhao X, Sun H, Xiong Z, Zhang D, Cen S, Wang J, Liang C, Guo F. 2019. MOV10 sequesters the RNP of influenza A virus in the cytoplasm and is antagonized by viral NS1 protein. Biochem J 476:467–481. doi:10.1042/BCJ20180754 30617221

[44] Haas AL, Warms JV, Hershko A, Rose IA. 1982. Ubiquitin-activating enzyme. Mechanism and role in protein-ubiquitin conjugation. J Biol Chem 257:2543–2548. doi:10.1016/S0021-9258(18)34958-5 6277905

[45] Mizushima N, Yoshimori T, Ohsumi Y. 2011. The role of Atg proteins in autophagosome formation. Annu Rev Cell Dev Biol 27:107–132. doi:10.1146/annurev-cellbio-092910-154005 21801009

[46] Yuan J, Shaham S, Ledoux S, Ellis HM, Horvitz HR. 1993. The C. elegans cell death gene ced-3 encodes a protein similar to mammalian interleukin-1 beta-converting enzyme. Cell 75:641–652. doi:10.1016/0092-8674(93)90485-9 8242740

[47] Zhang J, Huang F, Tan L, Bai C, Chen B, Liu J, Liang J, Liu C, Zhang S, Lu G, Chen Y, Zhang H. 2016. Host protein moloney leukemia virus 10 (MOV10) acts as a restriction factor of influenza A virus by inhibiting the nuclear import of the viral nucleoprotein. J Virol 90:3966–3980. doi:10.1128/JVI.03137-15 PMC4810528 26842467

[48] Mo Q, Xu Z, Deng F, Wang H, Ning YJ. 2020. Host restriction of emerging high-pathogenic bunyaviruses via MOV10 by targeting viral nucleoprotein and blocking ribonucleoprotein assembly. PLoS Pathog 16:e1009129. doi:10.1371/journal.ppat.1009129 PMC7746268 33284835

[49] Burdick R, Smith JL, Chaipan C, Friew Y, Chen J, Venkatachari NJ, Delviks-Frankenberry KA, Hu WS, Pathak VK. 2010. P body-associated protein Mov10 inhibits HIV-1 replication at multiple stages. J Virol 84:10241–10253. doi:10.1128/JVI.00585-10 PMC2937795 20668078

[50] Furtak V, Mulky A, Rawlings SA, Kozhaya L, Lee K, Kewalramani VN, Unutmaz D. 2010. Perturbation of the P-body component Mov10 inhibits HIV-1 infectivity. PLoS One 5:e9081. doi:10.1371/journal.pone.0009081 PMC2816699 20140200

[51] Huang F, Zhang J, Zhang Y, Geng G, Liang J, Li Y, Chen J, Liu C, Zhang H. 2015. RNA helicase MOV10 functions as a co-factor of HIV-1 Rev to facilitate Rev/RRE-dependent nuclear export of viral mRNAs. Virology 486:15–26. doi:10.1016/j.virol.2015.08.026 26379090

[52] Puray-Chavez MN, Farghali MH, Yapo V, Huber AD, Liu D, Ndongwe TP, Casey MC, Laughlin TG, Hannink M, Tedbury PR, Sarafianos SG. 2019. Effects of moloney leukemia virus 10 protein on hepatitis B virus infection and viral replication. Viruses 11:651. doi:10.3390/v11070651 PMC6669478 31319455

[53] Ariumi Y. 2022. Host cellular RNA helicases regulate SARS-CoV-2 infection. J Virol 96:e0000222. doi:10.1128/jvi.00002-22 PMC8941876 35107372

[54] Yang X, Xiang Z, Sun Z, Ji F, Ren K, Pan D. 2022. Host MOV10 is induced to restrict herpes simplex virus 1 lytic infection by promoting type I interferon response. PLoS Pathog 18:e1010301. doi:10.1371/journal.ppat.1010301 PMC8880913 35157734

[55] Zhang J, Zhang L, Liu D, Shi H, Zhang X, Chen J, Yang X, Zeng M, Zhang J, Feng T, Zhu X, Jing Z, Ji Z, Shi D, Feng L. 2024. Helicase protein DDX11 as a novel antiviral factor promoting RIG-I-MAVS-mediated signaling pathway. mBio 15:e0202824. doi:10.1128/mbio.02028-24 PMC11633105 39470258

[56] Heaton SM, Borg NA, Dixit VM. 2016. Ubiquitin in the activation and attenuation of innate antiviral immunity. J Exp Med 213:1–13. doi:10.1084/jem.20151531 PMC4710203 26712804

[57] Song L, Luo ZQ. 2019. Post-translational regulation of ubiquitin signaling. J Cell Biol 218:1776–1786. doi:10.1083/jcb.201902074 PMC6548142 31000580

[58] Li F, Li Y, Liang H, Xu T, Kong Y, Huang M, Xiao J, Chen X, Xia H, Wu Y, Zhou Z, Guo X, Hu C, Yang C, Cheng X, Chen C, Qi X. 2018. HECTD3 mediates TRAF3 polyubiquitination and type I interferon induction during bacterial infection. J Clin Invest 128:4148–4162. doi:10.1172/JCI120406 PMC6118574 29920190

[59] Sun N, Jiang L, Ye M, Wang Y, Wang G, Wan X, Zhao Y, Wen X, Liang L, Ma S, Liu L, Bu Z, Chen H, Li C. 2020. TRIM35 mediates protection against influenza infection by activating TRAF3 and degrading viral PB2. Protein Cell 11:894–914. doi:10.1007/s13238-020-00734-6 PMC7719147 32562145

[60] Gao P, Ma X, Yuan M, Yi Y, Liu G, Wen M, Jiang W, Ji R, Zhu L, Tang Z, Yu Q, Xu J, Yang R, Xia S, Yang M, Pan J, Yuan H, An H. 2021. E3 ligase Nedd4l promotes antiviral innate immunity by catalyzing K29-linked cysteine ubiquitination of TRAF3. Nat Commun 12:1194. doi:10.1038/s41467-021-21456-1 PMC7895832 33608556

[61] Chen HW, Yang YK, Xu H, Yang WW, Zhai ZH, Chen DY. 2015. Ring finger protein 166 potentiates RNA virus-induced interferon-β production via enhancing the ubiquitination of TRAF3 and TRAF6. Sci Rep 5:14770. doi:10.1038/srep14770 PMC4600972 26456228

[62] Kim S, Park K, Oh JM, Kim H. 2021. RNF126 is a positive regulator of TRAF3 ubiquitination. Biosci Biotechnol Biochem 85:2420–2428. doi:10.1093/bbb/zbab177 34643674

[63] Beachboard DC, Horner SM. 2016. Innate immune evasion strategies of DNA and RNA viruses. Curr Opin Microbiol 32:113–119. doi:10.1016/j.mib.2016.05.015 PMC4983539 27288760

[64] Isaacson MK, Ploegh HL. 2009. Ubiquitination, ubiquitin-like modifiers, and deubiquitination in viral infection. Cell Host Microbe 5:559–570. doi:10.1016/j.chom.2009.05.012 PMC7103382 19527883

[65] Zhong B, Zhang Y, Tan B, Liu TT, Wang YY, Shu HB. 2010. The E3 ubiquitin ligase RNF5 targets virus-induced signaling adaptor for ubiquitination and degradation. J Immunol 184:6249–6255. doi:10.4049/jimmunol.0903748 20483786

[66] Mahon C, Krogan NJ, Craik CS, Pick E. 2014. Cullin E3 ligases and their rewiring by viral factors. Biomolecules 4:897–930. doi:10.3390/biom4040897 PMC4279162 25314029

[67] Han J, Kim KH, Seo SU. 2025. The ubiquitin-proteasome system in hepatitis B virus infection and hepatocarcinogenesis: viral manipulation and therapeutic targets. J Microbiol Biotechnol 35:e2508005. doi:10.4014/jmb.2508.08005 PMC12615137 41162171

[68] Han Y, Zhang J, Shi H, Zhou L, Chen J, Zhang X, Liu J, Zhang J, Wang X, Ji Z, Jing Z, Cong G, Ma J, Shi D, Li F. 2019. Epitope mapping and cellular localization of swine acute diarrhea syndrome coronavirus nucleocapsid protein using a novel monoclonal antibody. Virus Res 273:197752. doi:10.1016/j.virusres.2019.197752 PMC7114574 31518629

[69] Xing Y, Chen J, Tu J, Zhang B, Chen X, Shi H, Baker SC, Feng L, Chen Z. 2013. The papain-like protease of porcine epidemic diarrhea virus negatively regulates type I interferon pathway by acting as a viral deubiquitinase. J Gen Virol 94:1554–1567. doi:10.1099/vir.0.051169-0 PMC4811637 23596270

[70] Liu D, Zeng M, Zhang J, Zhang L, Shi H, Zhang X, Zhang J, Chen J, Ji Z, Li X, Gu G, Feng T, Shi D, Sun D, Feng L. 2025. The swine acute diarrhea syndrome coronavirus spike protein promotes syncytial formation via upregulation of cellular cholesterol synthesis. mBio 16:e00976-25. doi:10.1128/mbio.00976-25 PMC12345205 40586601

[71] Bustin SA, Benes V, Garson JA, Hellemans J, Huggett J, Kubista M, Mueller R, Nolan T, Pfaffl MW, Shipley GL, Vandesompele J, Wittwer CT. 2009. The MIQE guidelines: minimum information for publication of quantitative real-time PCR experiments. Clin Chem 55:611–622. doi:10.1373/clinchem.2008.112797 19246619

