# Supplementary material for: MOV10 inhibits SADS-CoV replication by enhancing TRIM24-mediated K63-linked TRAF3 ubiquitination, and this inhibition is antagonized by viral N protein
Source: J Virol. 2026 Sep 29;100(10):e01215-26. doi: 10.1128/jvi.01215-26 (PMC13712983; doi:10.1128/jvi.01215-26)
Supplement: Supplementary file 1 [file jvi.01215-26-s0005.docx]

**Figure S1.** (A) MOV10 interacted with the SADS-CoV N protein. HEK293T cells were transfected or co-transfected with plasmids encoding Myc-hMOV10 and GFP-N. Cell lysates were subjected to immunoprecipitation using GFP Abs. (B) HEK293T cells were infected with SADS-CoV (MOI = 0.1) for 24 h. Lysates were subjected to immunoprecipitation using MOV10 Ab, with IgG Ab as the negative control. Immunoprecipitants were analyzed by western blotting using Abs against MOV10, SADS-CoV N, and GAPDH. (C) Schematic of full-length SADS-CoV N and its serial truncated mutants. (D and E) MOV10 interacted with N1 and N2 domains of SADS-CoV N protein. HEK293T cells were transfected or co-transfected with plasmids encoding HA-pMOV10, GFP-N, GFP-N1, GFP-N2, or GFP-N3. Cellular lysates were subjected to immunoprecipitation using HA (D) or GFP (E) Abs. (F) Interaction between SADS-CoV N and MOV10 was likely RNA-dependent. Plasmids encoding Myc-hMOV10 and GFP-N were transfected or co-transfected into HEK293T cells in the presence or absence of RNase A (100 µg/mL). Cell lysates were subjected to immunoprecipitation using Myc Abs. The density of SADS-CoV N protein bands relative to GAPDH was calculated, with the values of each negative control group normalized to 1. (G-I) The N-terminus of MOV10 was required for inhibition of SADS-CoV replication. ST cells were transfected with HA-pMOV10 or its truncations (HA-pMOV10-N and HA-pMOV10-C) for 24 h, followed by infection with SADS-CoV (MOI = 0.1) for an additional 24 h. Cell lysates were analyzed by western blotting (G). The density of SADS-CoV N bands relative to GAPDH was calculated using grayscale analysis. SADS-CoV N gene RNA levels were quantified by RT-qPCR (H). Viral titers were determined using TCID_50_ assays (I). The mean and SD of the results from three independent experiments are shown (**P* < 0.05; ***P* < 0.01; ****P* < 0.001).

**Figure S2.** (A and B) siRNA-mediated MOV10 knockdown experiment. IPI-2I cells were transfected with siRNAs (siMOV10-1, siMOV10-2 and siMOV10-3) or siNC at 50 nM for 48 h. Western blotting (A) and RT-qPCR (B) assessed the MOV10 knockdown efficiency. Band density for MOV10/GAPDH was calculated, with values from the siNC group standardized to 1. (C and D) siRNA-mediated TRIM24 knockdown experiment. HEK293T cells were transfected with three siRNAs against porcine TRIM24 and siNC at 50 nM for 48 h. Western blotting (C) and RT-qPCR (D) assessed expression of TRIM24. Band density for MOV10/GAPDH was calculated, with values from the siNC group standardized to 1. The mean and SD of the results from three independent experiments are shown (**P* < 0.05).

**Figure S3.** (A) Schematic of CRISPR/Cas9-based knockout cell line construction. (B and C) Expression of MOV10 in WT and MOV10^−/−^ IPI-2I cells were assessed by western blotting and RT-qPCR. (D and E) Expression of TRIM24 in WT and TRIM24^−/−^ HEK293T cells was assessed by western blotting and RT-qPCR. The mean and SD of the results from three independent experiments are shown (**P* < 0.05).

**Figure S4.** (A) Endogenous MOV10 interacted with TRIM24. HEK293T cells were harvested for Co-IP using MOV10 Ab, with IgG Ab as the negative control. Immunoprecipitants were analyzed by western blotting using Abs against MOV10, TRIM24, and GAPDH. (B) HEK293T cells were co-transfected with plasmids encoding Flag-TRAF3, GFP-N, GFP-N1, GFP-N2, or GFP-N3. Cellular lysates were subjected to immunoprecipitation using Flag Ab. (C) Plasmids encoding TRAF3, DelRING mutant, DelZINC mutant, DelCC mutant, and DelMATH mutant containing Flag tag were co-transfected into HEK293T cells with Myc-N, respectively. Lysates were subjected to immunoprecipitation using Flag Ab. (D) HEK293T cells were co-transfected with plasmids Flag-TRAF3 MATH and GFP-N2. Lysates were subjected to immunoprecipitation using Flag Ab. (E) Co-localization of TRAF3 and SADS-CoV N protein. Plasmids expressing Flag-TRAF3 (red) and Myc-N (green) were transfected or co-transfected into Vero E6 cells. Merged images show co-localization of these proteins. Nuclei are highlighted by DAPI staining (blue). (Bar: 5 µm). (F) HEK293T cells were transfected with plasmids encoding Myc-N or EV. Lysates were subjected to immunoprecipitation using MOV10 Ab, with IgG Ab as the negative control. (G) IPI-2I cells were treated with 20 μM CQ, MG132, or Z-VAD-FMK for 24 h. DMSO-treated cells were used as a control group. Cytotoxicity was detected by CCK-8 assay.
